# Study on the Influence of Polymer/Particle Properties on the Resilience of Superhydrophobic Coatings

**DOI:** 10.1021/acsomega.2c01547

**Published:** 2022-05-18

**Authors:** Yasmin A. Mehanna, Colin R. Crick

**Affiliations:** † Materials Innovation Factory, Department of Chemistry, University of Liverpool, Liverpool L69 7ZD, U.K.; ‡ School of Engineering and Materials Science, Queen Mary University of London, Mile End Road, London E1 4NS, U.K.

## Abstract

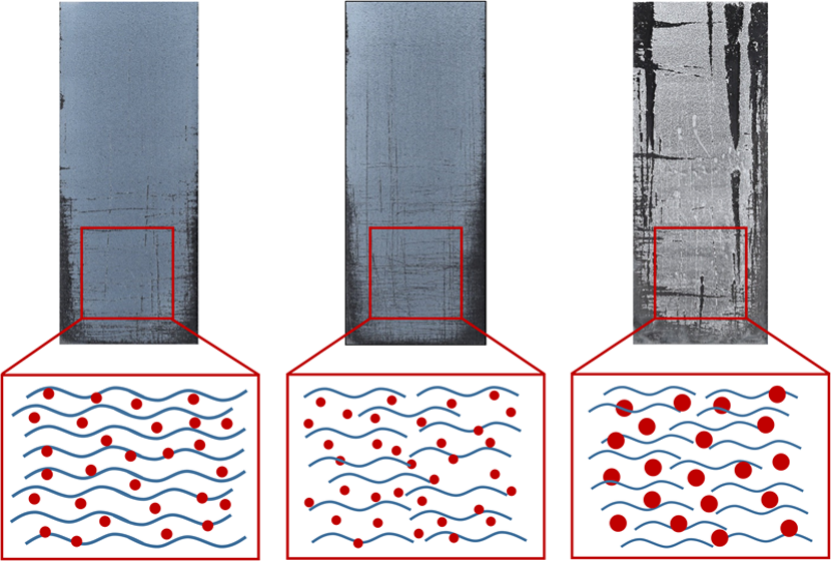

Enhancement in the resilience of superhydrophobic coatings is crucial for their future applicability. However, the progress in this aspect is currently limited due to the lack of a consistent resilience analysis methodology/protocol as well as the limited understanding of the influence of the materials components on the resultant coating performance. This study applies a quantitative analysis methodology involving image analysis and mass tracking and utilizes it to investigate how the properties of coating components can influence coating resilience. The factors examined were changing the molecular weight/tensile strength of poly(vinylchloride)/poly(dimethylsiloxane) (PVC/PDMS) polymers and changing the size of the roughening particles. In addition to the examination of resilience data to evaluate degradation patterns, three-dimensional (3D) mapping of the scratches was performed to obtain an insight into how material removal occurs during abrasion. The results can indicate preferential polymer selection (using higher-molecular-weight polymers for PVC) and optimal particle sizes (smaller particles) for maximizing coating resilience. The study, although focused on superhydrophobic materials, demonstrates wide applicability to a range of areas, particularly those focused on the development of high-strength coatings.

## 1 Introduction

The interest in superhydrophobic materials has been rapidly growing and widely expanding into different application fields.^1−3^ This is due to their ability to selectively repel water and hence enhance the performance of many systems whose applications involve a high degree of exposure to water.^4,5^ Examples include self-cleaning,^6,7^ drag reduction,^8,9^ oil/water separation,^10,11^ antifouling,^12,13^ in addition to others. For many superhydrophobic coatings, water repellence is maximized when materials with low surface energy possess a surface structure with a high roughness, including micro/nano textures.^14^ Rough micro/nanoscale surfaces are usually physically weak, as they are more prone to physical degradation in comparison to smooth/flatter surfaces.^14^ Routes to fabricating robust superhydrophobic materials, and developing a general approach for doing so are central challenges in this research area.

Many research reports have attempted to fabricate superhydrophobic coatings with high robustness. Wu et al. reported a fluorinated resin/Fe_3_O_4_ nanoparticle-based coating prepared by inverse infiltration, where a two-layer coating was prepared by spraying and curing of a base layer followed by spraying of the polymer/nanoparticle mixture.^15^ This allowed polymer infusion through both layers, which strengthened the adhesion of nanoparticles. The coatings maintain superhydrophobicity through harsh abrasion conditions (260 cycles of sandpaper abrasion and 25 cycles of sand impact). Deng et al. utilized porous silica capsules to make a superhydrophobic coating and incorporated chemical vapor deposition (CVD) to chemically bind the silica to enhance its resilience.^16^ It was shown, using sand impact and tape peeling tests, that CVD has significantly improved superhydrophobicity retainment compared to where the capsules are binding only by weak van der Waals interactions. Kondrashov et al. generated hierarchical micro-cones/nano-grass silicon surfaces using a dry etching process.^17^ By optimization of the micro-cone density, apex angle, and length, the surface was able to retain superhydrophobicity after 20 N shear load. These approaches generally focus on the generation of a material/coating with a consistent binding force throughout the structure.

While attempts for fabricating robust superhydrophobic coatings are numerous, systematic progress toward truly resilient materials is limited. This can be rationalized due to first the lack of consistent degradation analysis protocol that enables direct comparison between different coatings reported, and second the lack of understanding of the source of robustness and how it is related to (and affected by) the properties of the materials forming the coating. Currently, many well-established abrasion methods have been utilized in the examination of coating resilience; this includes sandpaper abrasion,^18,19^ pensile hardness,^20,21^ nano-indenters,^22,23^ tape peeling,^24,25^ and sand impact^26,27^—in addition to others. Despite the frequent adoption of these techniques, the specific protocol utilized can greatly vary between different reports, including the definition of an abrasion cycle and the load applied on the coating. This divergence makes deducing definitive conclusions and planning routes in the development of resilient coatings extremely challenging. Furthermore, many of these reports include composite materials, which presents an additional layer of complexity when considering how each component may influence and contribute to the robustness of the coating. This in-depth consideration is not normally reported, and in combination with the lack of consistency in analysis techniques hinders the progress of research efforts.

A straightforward robustness analysis methodology was reported previously by the authors, which utilizes a combination of image processing and mass tracking for abraded coatings.^28^ In this technique, sandpaper abrasion is conducted to a surface, and images are taken by a conventional electronic scanner after each abrasion cycle. A postimaging analysis is carried out using MATLAB, and a code designed to convert scanned images into binary (black/white pixels represent coating remained/coating removed, respectively) and to calculate the percentage of remaining/undamaged coating. In addition, to complement this analysis, the change in coating mass is recorded. This technique allows quantitative analysis of the amount of coating that persisted after each abrasion cycle and also deducing the relative rate/ease of the coating degradation. In addition, comparing the percentage of coating remaining as predicted by both mass tracking and image analysis allows differentiation between adhesion and cohesion failures of the material being considered. This is a result of the “weighing” approach detecting the entire amount of coating removed, while the “imaging” approach only detects visible scratches and not superficial coating removal.

In the current study, the purpose is to utilize this quantitative degradation methodology as a tool to evaluate the resilience of superhydrophobic polymer/particle composite (SPPC) coatings^29^ and understand how the components’ properties contribute to the resultant resilience. While the previous study outlined the technique and explained its main principles, this study investigates the applicability of this technique while focusing on a different aspect, at the same time improving the resilience of SPPC coatings. These coatings utilize a relatively simple three-component formulation (solvent, polymer, and micro/nanoparticle). The effect of varying the properties of the composite formulation components has not been reported in the literature. The main factors examined here are the effect of (i) variation in the physical properties of the polymer (*M*_w_, tensile strength) and (ii) changing the particle size and size distribution.

## 2 Experimental Section

### 2.1 Materials

Poly(vinylchloride) (PVC) was purchased from Sigma-Aldrich with molecular weights (*M*_w_, as reported by the manufacturer) of 48,000, 90,000, and 233,000 Daltons, respectively (product number 81388, product number 81387, and product number 346764). These will be referred to as PVC-L, PVC-M, and PVC-H for low, medium, and high *M*_w_, respectively. Likewise, poly(dimethylsiloxane) (PDMS) was purchased in three different forms. Sylgard 186 Silicone Elastomer (a two-part thermosetting PDMS elastomer, catalyzed with a platinum curing agent) was purchased from Ellsworth Adhesives Ltd. Two further silicone elastomers in the same product line (Sylgard 182 and Sylgard 184) were purchased from Dow. These elastomers differ (along with other properties) in their tensile strength (TS) as reported by the manufacturer (2.1, 6.7, and 7.6 N/mm^2^) for Sylgard 186, Sylgard 184, and Sylgard 182, respectively. These are referred to as PDMS(186), PDMS(184), and PDMS(182), respectively.

Silicon dioxide nanopowder (Ø—10–20 nm) and hexamethyldisilazane (HMDS, reagent grade, ≥99%) were purchased from Sigma-Aldrich. Silicon dioxide powder (Ø ∼ 1.5 μm, 99.9%) was purchased from Alfa Aesar. Hexane (HPLC grade), tetrahydrofuran (THF, ≥99.5%, laboratory reagent grade), and toluene (≥99.8%) were purchased from Fisher Scientific Limited. Glass microscope slides purchased from Thermo Scientific were used as the substrates. An adhesion promoter (CYN20 Stick 2 Industrial Grade General Purpose Adhesive—cyanoacrylate based) was purchased from EverBuild. Sandpaper sheets (grit no. 120, dimensions; 23 × 9 cm^2^) were purchased from Miady.

### 2.2 Silica Hydrophobization

A similar procedure was followed for both nano-sized (referred to as nSiO_2_) and micron-sized (referred to as μSiO_2_) silicon dioxide powder, which was detailed in previous work.^28^ A solution of HMDS (1 mL) in toluene (100 mL) was added to a suspension of as silica (10 g) in toluene (250 mL) and refluxed at 120 °C for 24 h with magnetic stirring. The hydrophobized particles were centrifuged, washed twice with toluene and a further two times with ethanol, subsequently dried at 90 °C overnight, and stored dry under ambient conditions.

### 2.3 Coating Preparation

For nSiO_2_, the relative ratios of polymer/silica/solvent in their respective formulation were investigated to achieve optimized superhydrophobicity (detailed in previous work).^29^ The previously reported ratios were applied here for both the PVC and PDMS coatings. The nSiO_2_/PVC coating solutions were prepared by dissolving PVC (0.1 g) in THF (30 mL) by stirring until fully dissolved (typically ∼15 min). Hydrophobized silica nanoparticles (0.235 g, polymer/silica mass ratio (*M*_ratio_) = 0.426) were then added and the mixture was stirred for 4 h to ensure complete polymer/nanoparticle mixing.

PDMS coating solutions were prepared by mixing both parts of the elastomer as recommended by the manufacturer (with a ratio of 10:1, total polymer mass = 1.086 g) with hexane (150 mL) and stirring until dissolved. This stock polymer solution was used (i) to prepare PDMS/SiO_2_ solutions (described below) and (ii) with the adhesive to make a precoating layer (see Section 2.4).

Hydrophobized nSiO_2_ (0.259 g, *M*_ratio_ = 1.4) was added to 50 mL of the PDMS stock solution and stirred at room temperature (RT) for an hour (note: this stirring time was reduced compared to the PVC solutions (above) to prevent the premature onset of the thermosetting reaction).

μSiO_2_ was utilized in combination with PDMS only, as superhydrophobic coatings could not be successfully formulated using PVC. Different *M*_ratio_ were tested, and the optimum ratio was found to be 0.3 (Figure SI1). Typically, 17 mL of the PDMS stock solution was diluted with 73 mL of hexane to provide a similar silica concentration. Hydrophobized μSiO_2_ (0.403 g) was added and stirred at room temperature for an hour.

A mixture of both nSiO_2_ and μSiO_2_ was also prepared. Typically, 30 mL of the PDMS stock solution was diluted with 20 mL of hexane, and then nSiO_2_ (0.129 g) and μSiO_2_ (0.121 g) were added and stirred at room temperature for an hour.

### 2.4 Coating Deposition

**Figure 1:**
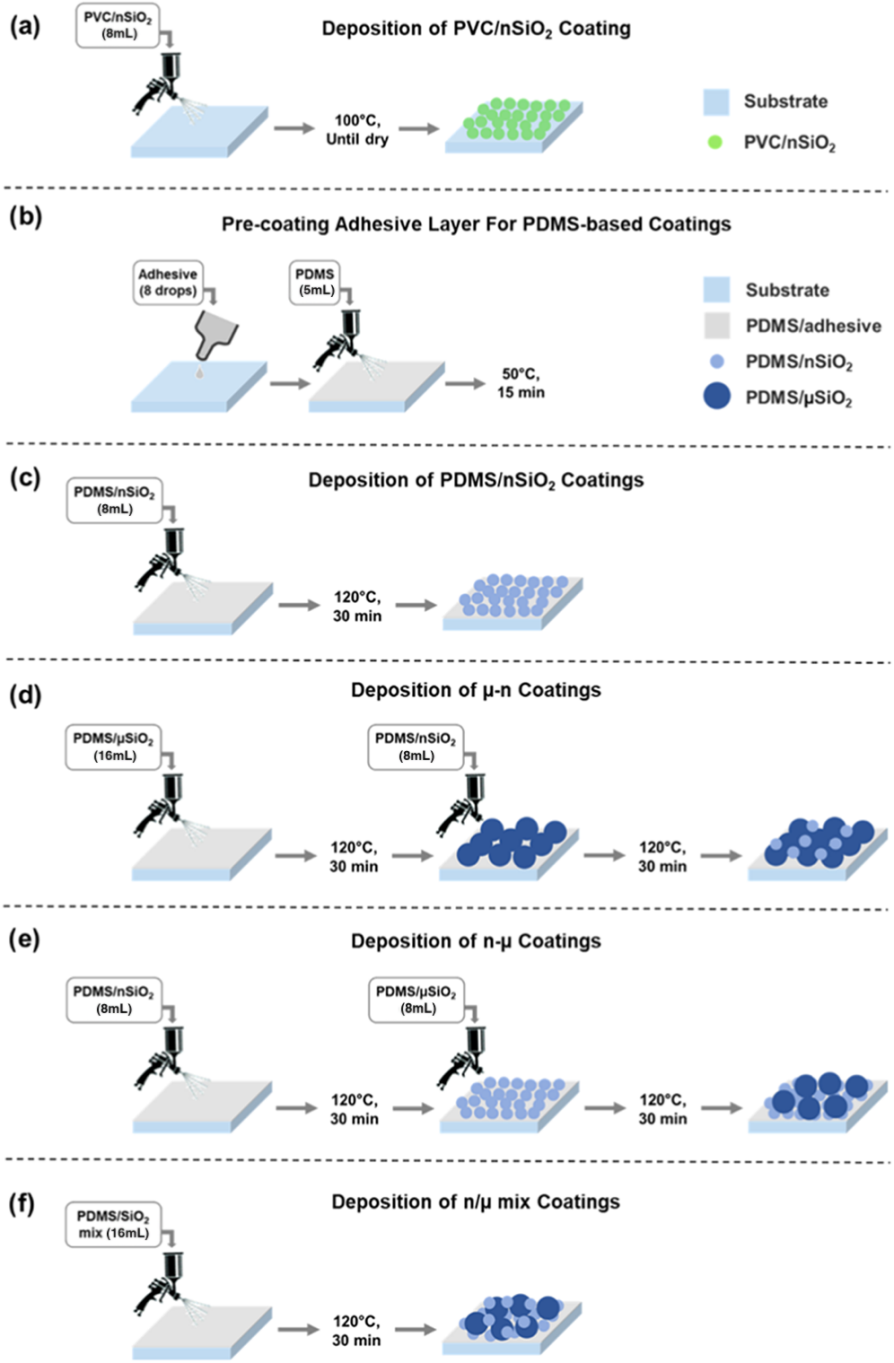
Schematic showing the coating deposition procedure for (a) PVC/nSiO_2_ coatings, (b) precoating adhesive layer for PDMS-based coatings, (c) PDMS/nSiO_2_ coatings, (d) μ–n coating, (e) n−μ coating, and (f) n/μ mix coating.

The spray coating process utilized in this study is illustrated in Figure 1 and was based on a previously reported methodology,^29,30^ using a compression pump and airbrush gun (manufactured by Voilamart), at a pressure of 2 bar. The coating suspensions (from Section 2.3) were sprayed onto glass substrates, with all spraying carried out ∼4 cm away from the surface to ensure a consistent substrate coverage.

For PVC coatings, glass slides were preheated at 100 °C for approximately 30 min, ensuring consistent heat across the substrate, followed by spraying ∼8 mL of the solution onto each glass slide at the same temperature. Coated slides were left for a few seconds until visibly dried (Figure 1a).

All PDMS coatings involved the precoating of substrates with an adhesive layer to promote higher robustness of the coatings. The conditions for this layer were kept similar across the different silica sizes/combinations examined. Typically, eight drops (∼150 μL) of the adhesion promoter (CYN20) were manually spread over the slide (using the edge of a microscope slide), followed by spraying approximately 4–5 mL of the PDMS stock solution. This was allowed to partially cure by heating at 50 °C for 15 min (Figure 1b).

For PDMS/nSiO_2_ coatings, the precoated slides were moved to a 120 °C-adjusted hotplate to spray ∼8 mL of the PDMS/silica solution. The coated slides were allowed to fully cure on a 120 °C hotplate for 30 min (Figure 1c).

The combination of different silica particle sizes was examined. For the two sizes included in this study, three combinations were made (Figure 1d–f): (i) a two-layer coating consisting of a PDMS/μSiO_2_ layer followed by a PDMS/nSiO_2_ layer (referred to as μ–n coating), (ii) a two-layer coating consisting of a PDMS/nSiO_2_ layer followed by a PDMS/μSiO_2_ layer (referred to as n−μ coating), and (iii) a single-layer coating: spraying a mixture of both nSiO_2_ and μSiO_2_ (as described in Section 2.3, referred to as n/μ mix). The first layer of the μ–n coating was prepared by spraying 16 mL of PDMS/μSiO_2_ solution, followed by curing at 120 °C for 30 min. The second layer was prepared by spraying 8 mL of PDMS/nSiO_2_ solution, followed by the same curing conditions (Figure 1d). For the n−μ coating, 8 mL of PDMS/μSiO_2_ solution was sprayed on top of an 8 mL PDMS/nSiO_2_ layer, and each layer was allowed to cure similarly (Figure 1e). Finally, the n/μ mix coating was made by spraying 16 mL of the mixture solution and curing as previously described (Figure 1f).

### 2.5 Resilience Assessment

The resilience assessment methodology was developed in a previous report.^28^ The process involved (i) sample abrasion, (ii) mass-loss tracking, and (iii) sample imaging (with subsequent image analysis). This process was repeated three times for each coating variation. Primarily, sandpaper abrasion was carried out, whereby the coated glass slide was placed face-down onto sandpaper (grit no. 120) with a 100 g weight placed on top of it. Both the glass slide and weight were pushed for 10 cm, before being turned 90° and moved a further 10 cm to complete one cycle (Figure SI2a).^29,31^ Mass of the coatings was continually assessed by weighing all glass slides before and after coating, as well as after each abrasion cycle, thus providing the mass loss during the abrasion process. The weight at each stage was measured twice (or until getting two readings with a maximum difference of less than 0.0005 g), with the average taken to ensure precision. After each cycle (as well as before abrasion at the 0 cycle), the coated slide was also scanned with an Epson Perfection V39 scanner (resolution used: 600 dpi), using a black paper card as backing to ensure a dark background for high contrast in the scanned images (Figure SI2b). All images were converted to binary using MATLAB to extract the percentage of the remaining coating (as outlined in the previous report).^28^

The assessment methodology offers tremendous flexibility in the materials analysis, whereby the abrasion methodology can be completely substituted for an alternative. In addition, the sensitivity of the analysis can be optimized for a range of substrates, including the separate consideration of red, green, and blue data from the RGB values detected.

### 2.6 Mechanical Testing and Films Preparation

Tensile stress–strain curves of polymers were carried out using a universal testing Machine (SHIMADZU EZTest) with a crosshead rate (pulling speed) of 3 mm/min. The testing temperature was fixed at 25 °C using an air conditioner. Dog-bone samples were made into ISO 527-2/5A size (Figure SI3a).

PVC samples were prepared as illustrated in Figure SI4. PVC (0.3 g) was added to 30 mL of THF and stirred until dissolved. This solution was poured into a crystallization dish (Ø = 11.5 cm), covered by aluminum foil (small holes were made to allow slow evaporation), and placed in a fume cupboard at RT. This was repeated every 4–6 h for a total of five iterations, to increase the overall film thickness (total polymer mass and THF volume = 1.5 g and 150 mL, respectively). After complete evaporation (∼ 48 h after the last solution was added), the film was removed from the dish and then cut into dog-bone-shaped pieces. Typically, a dog-bone-shaped metal cutter was heated at 110 °C, placed on a PVC film, and put in a hot press (heated at the same temperature). The press was secured and a pressure of around 1.5 MPa was applied (indicated by a pressure gauge attached to the press). The film was quickly removed after 20 s and stored in ambient conditions until mechanical testing was carried out. Each film produced five to six dog-bone samples, which were all tested and the average of the closest three runs was obtained. Furthermore, three films were prepared for each polymer to make three testing rounds; hence, the reported averages here (Section 3.1) are for nine runs.

PDMS samples were prepared according to a previous report.^32^ The two Sylgard 184 components (PDMS = 5 g, ratio 10:1 PDMS to curing agent) were magnetically stirred at 200 rpm for 30 min. The mixture was then moved into a vacuum desiccator for 30 min to remove air bubbles. The three-part mold consisted of two top and bottom aluminum sheets (covered with grease paper to facilitate sample removal) and a three-dimensional (3D)-printed 100 × 40 × 2 mm^2^ piece of tough polylactic acid (PLA) in the middle with a hole of ISO 527-2/5A dimensions (Figure SI3b). The PLA part was placed above the covered aluminum sheet, and the polymer was poured inside the hole, and then covered with the other grease paper/aluminum sheet. The mold was then clamped and placed vertically for 30 min to ensure that any remaining bubbles will move upward away from the testing region. The sample was cured at 120 °C for 33 min and then removed from the mold once cooled down.

### 2.7 Characterization

A drop shape analyzer was used to measure WCAs, using a water droplet volume of 5 μL. This was repeated five times for each coating, and the average was calculated. Scanning electron microscopy (SEM) images were performed using a field emission microscope (JEOL, JSM-7001F) with an acceleration voltage of 3 kV.

Confocal fluorescence microscopy was carried out using a Zeiss LSM 880 upright confocal microscope on a Zeiss Axio Examiner Z1 (Zeiss, Jena, Germany) with a 20×/1.0 Dic (water immersion) objective (Zeiss). Samples were excited using laser lines diode (461 nm). Data were captured using ZEN software (Zeiss, Jena, Germany).

Fluorescent coatings were prepared in a similar procedure to that mentioned in Section 2.3. A stock solution of Nile Red dye in chloroform (1.96 mM, 10 mL) was prepared. Fluorescent PVC/nSiO_2_ coatings were made by adding 120 μL of the dye solution to a premade solution of PVC (0.1 g) in THF (30 mL). This was stirred for 30 min before adding the nSiO_2_ (0.235 g), and the following stirring and spraying conditions were kept the same as the previously described PVC/nSiO_2_ coatings (Section 2.3). Likewise, fluorescent PDMS/nSiO_2_ coatings were made by solvating PDMS (0.5068 g, 10:1 of polymer:curing agent) in 70 mL of hexane, then adding 290 μL of the dye solution and was stirred for 30 min. nSiO_2_ (0.2586 g) was added to 50 mL of the prepared solution and stirred for an hour, while the remaining solution was used for the adhesive layer, as described in Section 2.3. The coatings were abraded, and the scratches were imaged.

## 3 Results and Discussion

The development of SPNC formulations was deeply investigated in a previous report. In these formulations, the coating is hypothesized to form by the encapsulation of particles by the polymer. The thickness of the polymer is crucial to the hydrophobicity as well as the functional properties of the coating. A deficiency of the polymer results in a low physical resilience due to poor interparticle adhesion. On the other hand, excess of the polymer quantity kills the superhydrophobicity, as the polymer fills the porosity provided by the arrangement of the particles. A detailed examination of the appropriate *M*_ratio_ of polymer/particle leading to the optimum polymer thickness was conducted for PDMS and PVC coatings with nSiO_2_. While only one polymer variant was studied (PDMS(184) and PVC-M), utilizing the other variants was not found to require readjustment of *M*_ratio_, as illustrated in Figure SI5, by the high WCAs achieved for the other polymer variants. This was not the case for the inclusion of μSiO_2_, where polymer optimization was conducted and the optimum *M*_ratio_ was found to be 0.3 (Figure SI1). Meanwhile, the applied combinations of n/μSiO_2_ (Section 3.3) were also found to be superhydrophobic (Figure SI5).

### 3.1 Mechanical Properties of Polymers

The resilience of a coating involves different aspects, which influence how the coating behaves under abrasion. These include the coating–substrate adhesion, as well as the cohesive forces within the coating material. While the former is expected to be affected by different parameters, the latter is mainly dominated by the component properties, including their mechanical strength. Therefore, it is important to know the mechanical properties of the polymers reported in this study before discussing the abrasion experiments. The PDMS polymers used in this study (discussed below) have been examined by the manufacturers; however, the physical resilience of the PVC polymers is not reported. As elaborated in the previous section, three different types of PVC and PDMS polymers, respectively, were investigated. The three PVC polymers differ in their *M*_w_, and it is expected that higher molecular weight results in an increased polymer strength due to the higher degree of intermolecular interactions between the polymeric chains.^33^ However, this has not been previously verified for these PVC polymers. Therefore, mechanical testing was conducted to obtain values for TS, strain at the breaking point, and elastic modulus (shown in Figure SI6). It was observed that the repetitions from different runs showed a high degree of variation that provided a high degree of error to these measurements, particularly in the elastic modulus values. This was most likely due to variation in the films introduced during their preparation method, e.g., the speed of solvent evaporation caused by the air circulation. The elastic modulus is calculated from the slope of the linear portion (elastic deformation) of the stress–strain curve, which tended to be very short with the polymer samples deforming mostly inelastically. This type of deformation mechanism maximizes the influence of any differences between the samples.^34,35^ For the strain values, this variation, although present, was less dramatic. It is noted that the maximum strain increases with increasing *M*_w_, which adheres to the previously stated expectations. Meanwhile, the most important to the study context is the TS values. It is observed that the values are relatively close to each other, especially when the error is taken into consideration. However, the average TS values are larger for higher *M*_w_’s, which is generally expected for thermoplastic polymers,^33^ while this variation is not particularly large (as seen in Figure SI6).

For PDMS polymers, the TS was provided by the supplier; however, these tests were carried out at a reported crosshead rate of 508 mm/min (details from the supplier). This is a much higher rate than that applied for the PVC films described above. To establish a reasonable comparison, predicting the TS at a lower crosshead rate was required. Generally higher strain rates, while decreasing the strain at which the sample breaks, increase the measured TS value.^36,37^ This is supported by another reported tensile test on Sylgard 184 at a different crosshead rate (5.13 N/mm^2^ at 254 mm/min by Johnston et al.).^32^ To confirm this, mechanical testing was conducted on a sample of Sylgard 184, and the TS was found to be around 1.7 N/mm^2^. The main note to take from that is, for the polymers tested in this study, PVC polymers show higher strength compared to the PDMS polymers.

### 3.2 Influence of Polymer

**Figure 2:**
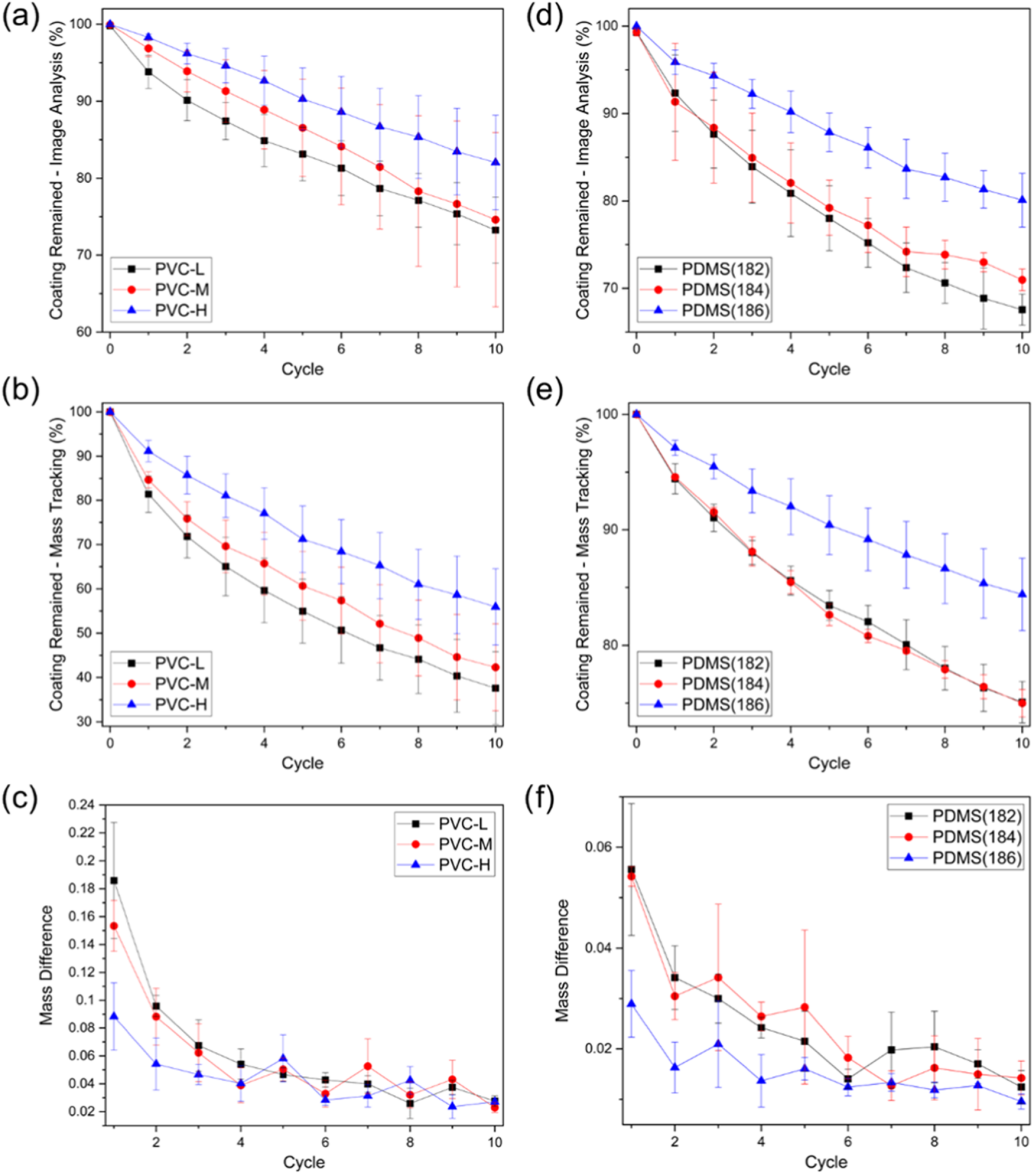
Plots for PVC (a–c) and PDMS (d–f) sample runs resilience performance vs abrasion cycles. (a, d) Percentage of coating remained as predicted by image analysis, (b, e) percentage of coating remained as measured by mass tracking, and (c, f) mass difference between every two consecutive cycles (normalized by dividing by the initial coating mass).

For the coatings discussed in this section, the incorporated particles were kept the same (nSiO_2_), along with all of the coating preparation and deposition conditions (detailed in Sections 2.3 and 2.4), while only the polymer was changed. As highlighted in the Section 1, both image processing and mass tracking were conducted for abraded coatings. The mass-loss data were expressed in two different ways: (i) as a percentage of the original coating weight (referred to as the percentage of coating remained by mass tracking) and (ii) by comparing the mass loss in each cycle (obtained by comparing the coating mass between two consecutive cycles, normalized by dividing by the original coating mass, referred to as mass difference). The former identifies the amount of coating that persisted after 10 abrasion cycles. The latter indicates the relative rate/ease of the coating degradation. The images from the abrasion experiment, along with the corresponding binary images and the estimated percentage of coating that remained, are shown in Figure SI7a–i for PVC coatings, and in Figure SI8a–i for PDMS coatings. Plots of percentages of coating remained by image analysis/mass tracking as well as the mass difference are shown in Figure 2a–c for PVC coatings, and in Figure 2d–f for PDMS coatings.

Comparing the degradation results for the PVC coatings, it can be noticed that PVC-H coatings tend to experience less damage as a result of abrasion. This is supported by image analysis (Figure 2a, percentage of coating remaining = 73, 75, and 82% for PVC-L, PVC-M, and PVC-H, respectively) as well as mass tracking results (Figure 2b, percentage of coating remaining = 38, 42, and 56% for PVC-L, PVC-M, and PVC-H, respectively). The mass difference with each abrasion cycle indicates a slower degradation process (i.e., lower mass loss between cycles) for PVC-H within the first few cycles (Figure 2c). This suggests that, while the three polymers tend to respond in a similar way as abrasion continues, the higher *M*_w_ (and, subsequently, higher TS) appears to minimize initial damage and delay the propagation of coating failure.

Further information could be extracted by comparing the percentage of coating remaining by mass tracking to that obtained by image analysis. This is beneficial as it provides additional insight into possible degradation pathways. As image analysis detects only visible scratches (and not minor scratches), while weighing is sensitive to all types of material removal. Observing the difference between image analysis and mass tracking data could provide an indication of superficial *vs* substantive coating removal (the former of which is detected by mass-loss tracking only). Utilizing this principle, the combined imaging/mass-loss analysis of the PVC samples demonstrates a significant difference in results achieved with the two approaches (Table 1), which suggests that superficial coating removal is taking place. This superficial failure is also following a similar pattern to that noticed with the percentages of coatings remaining (both by mass-loss and image analysis), as PVC-H appears to lose less coating mass as a result of this type of failure. This shows that the higher-*M*_w_ polymer is also more resistant to cohesion failure.

**Table 1:** Percentage of Coating Remained (Obtained by Mass Tracking and Image Analysis) for the Coatings Discussed^a^

coating	PVC-L (%)	PVC-M (%)	PVC-H (%)	PDMS(182) (%)	PDMS(184) (%)	PDMS(186) (%)	μ–n (%)	n–|μ (%)	n/|μ mix (%)
avg. % remained—by mass	37	43	56	75	75	84	59	71	65
avg. % remained—image analysis	73	75	82	67	71	80	55	75	56
difference	36	32	26	–8	–4	–4	–4	4	–9

a The difference (by image analysis—by mass) is also indicated.

**Figure 3:**
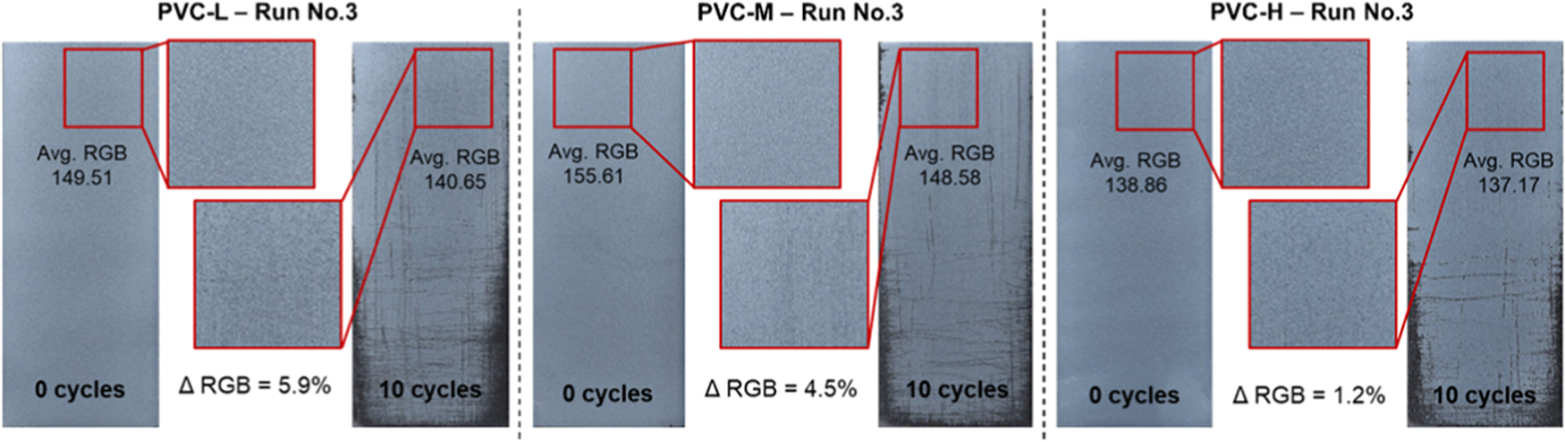
Comparison between superficial coating removal for PVC coatings. Regions with minimal visible scratches in 10_cycles images were compared to those in 0_cycles images in terms of RGB change (ΔRGB).

To demonstrate the previous deduction in another way, images for the three PVC polymers were taken after 10 abrasion cycles. A square area was cropped (similar dimensions for all samples) from a region where no/very little scratches are visible (Figure 3). The average RGB was calculated for these low damage areas and compared to the average RGB value of the same region before abrasion (cycle 0). While changes in RGB values would not be noticeable until substantive change occurs, a decrease in RGB value still indicated a partial coating removal. As the figure shows, this decrease was minimal for the PVC-H coating. The above conclusions could be justified by the increased intermolecular forces for longer polymer chains, and hence, the higher force required to separate/remove these chains.

**Figure 4:**
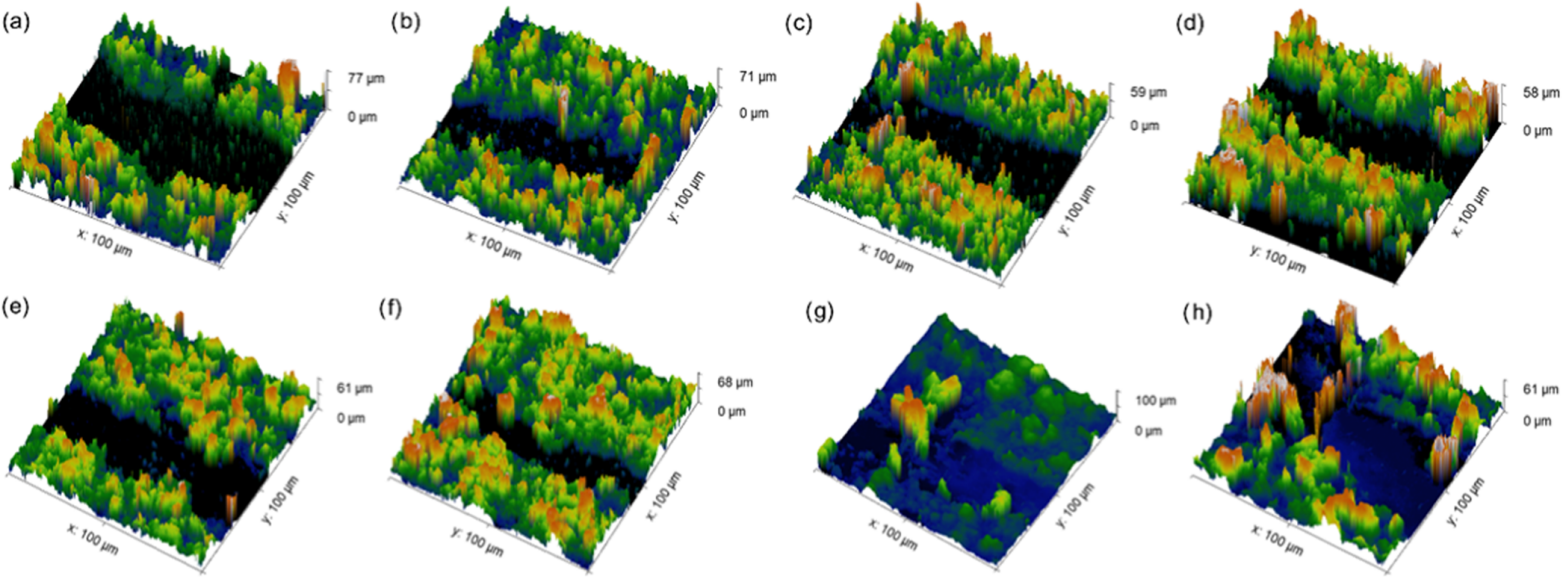
Confocal fluorescence images of scratches on abraded coatings of PVC-L (a, b), PVC-M (c, d), PVC-H (e, f), and PDMS(186) (g, h).

Confocal fluorescence microscopy was carried out to investigate the nature of the scratches in PVC coatings. Figure 4 shows a 3D mapping of 200 μm × 200 μm scratch areas on abraded coatings after 10 abrasion cycles. The coatings imaged were PVC-L (a, b), PVC-M (c, d), PVC-H (e, f), in addition to PDMS(186), which will be discussed later on in this section. The common observation in PVC scratch images is that the material removal does not affect the surrounding areas near the scratch, i.e., the coating height near the scratch is similar to the rest of the imaged area. This, again, indicates the low cohesion between the coating components in PVC coatings, as it is easily detached from the neighboring materials.

Abrasion analysis was carried out similarly for PDMS coatings. However, due to the PDMS polymers generally producing a less white coating (i.e., with lower RGB values) in comparison with PVC polymers (particularly for PDMS(182) and PDMS(184) coatings), some noise was noticed with the initial cycles (before abrasion). The relatively darker coating color originates from the higher transparency of PDMS and differences in morphology causing less light scattering. Ideally, the preabrasion images should provide a percentage value with no coating removed (i.e., 100% of the coating remaining). However, some of these initial images had lower percentages due to the noise present in the relatively darker starting material. This results in the percentages of the following cycles being slightly lower than expected. A correction was applied to account for this noise. This was made for sample runs with % coating for the zeroth cycle image <99% and was corrected to reach 99%. For instance, a sample run with a % coating for the zeroth cycle image = 94% means that there is 5% noise being read and deducted for all of the images in this set. Hence, a correction is made by adding 5% to the % coating values of the whole image set. This was judged to be reasonable, as this background noise could be tracked consistently in all images after abrasion and not just in the primary cycles. These image sets are shown in Figure SI8b–d,f (with the original percentages obtained by the analysis code), and the values before and after correction are shown in Figure SI9.

Regarding the performance of the PDMS polymers against abrasion, it can be noticed that both PDMS(182) and PDMS(184) follow similar trends in coating degradation, whereby the percentage of coating remaining (as measured by mass tracking) was 75% for both coating compositions. This can be rationalized through the relatively small difference in their TS values. The similar mass differences for these polymers between abrasion cycles suggest that the degradation initiation and propagation mechanisms are comparable. However, PDMS(186), which has a significantly lower TS value, showed higher resistance to coating removal (total % of coating remaining = 84%), as well as a slower degradation pattern. Image analysis supported this conclusion as well, with percentages of coating remaining were found to be 67, 71, and 80% for PDMS(182), PDMS(184), and PDMS(186), respectively.

It is noticed here that, unlike what was observed with PVC polymers, the abrasion resistance tended to increase with lower TS. To explain this, the differences between PVC and PDMS should be considered. PVC is a thermoplastic polymer, where the polymeric chains are only connected by weak intermolecular forces that do not fully restrict their relocation as a response to an outer stimulus, e.g., stress. It should also be noted that the major deformation mechanism for the PVC samples was inelastic. While the bonding between these chains can, in general, enhance the polymer performance, they can still break with moderate force. During the abrasion experiment, the sandpaper particles apply a certain amount of force that exceeds the binding force experienced by some of the polymer chains, inelastically deforming, and causing the polymer to be removed from the coating. Higher binding forces (indicated, in this case, by the increased M_w_ and tensile strength) lead to less polymer removal. In contrast, PDMS is a thermoset polymer where curing and covalent cross-linking result in a rigid network of polymer chains. Unlike thermoplastics, the degradation is expected to take place by the removal of larger bulks of polymers rather than loose chains, due to the stronger covalent binding and the large proportion of elastic deformation prior to breaking. While a low abrasion force would be expected to not cause much damage to such rigid networks, damage can still occur using a high enough force. The extent of damage would be related to the extent of cross-linking, with higher cross-linking meaning more connected areas that, when degradation is initiated in a part of it, complete removal of the whole network is expected. If the difference in TS for the PDMS polymers is reflected in the increased extent of cross-linking, then this could explain the behavior of PDMS(186). Although this justification explains the observed pattern in these polymer systems, it must be noted that the coatings explored here have a higher order of complexity than the mechanical testing of bulk polymers. The materials are nanocomposites, as such, their mechanical performance may vary from bulk measurements. Moreover, the reported PDMS-based coatings were applied using an adhesive layer which, although applied consistently throughout this study, could potentially affect the failure mechanism that would be observed for the basic polymer/particle composites. In addition, many parallels can be drawn between abrasion measurements and mechanical testing. However, these do have fundamental differences, the most notable of these is that abrasion testing also probes the effect of surface shear.

Another observation is that, while the TS for PDMS polymers is much lower compared to PVC polymers, the image analysis of coatings for both polymer types showed similarity in performance and even an enhancement in resistance in the PDMS polymers in terms of mass-loss tracking. This could be attributed again to the differences between them. Besides the relative degree of cross-linking in the PDMS/PVC polymers and the probable deviation of nanocomposites from the bulk polymer behavior, there are differences in their failure mechanism as indicated by their stress–strain curves (Figure SI10). For PVC, the test sample tended to record a maximum stress value shortly after the test starts, then the stress drops and the sample continues to elongate further (for 150–250% of its original length) while the stress increases, until recording another maximum just after the sample breaks. For PDMS, the strain increases steadily with the stress until reaching the breaking point. While a complete understanding of the implications of these differences in the coating resistance for abrasion is not complete, the main conclusion that could be extracted from this is that these differences invalidate direct comparisons between both types of polymers.

The final analysis to consider for PDMS results is the difference between mass-loss and image analysis values (Table 1). Here, these values were more comparable in contrast to the case with PVC coatings, suggesting that coating–substrate adhesion failure is dominant for these coatings (i.e., when the coating material is removed by abrasion, generally all underlying material is also removed). However, it is noted that the mass percentages were slightly higher than that obtained by image analysis for the PDMS coatings, resulting in a negative difference (−8% for PDMS(182), and −4% for both PDMS(184) and PDMS(186)). Theoretically, the mass values should be either lower than (as some coating removal is not detectable by imaging) or equal to the image analysis. This reversed behavior could be related to parts of the coating being detached from the substrate but not from the rest of the coating material, leaving small portions of the polymer.^28^ This is illustrated by the confocal images of scratches in a PDMS(186) coating (Figure 4g,h). In comparison to the PVC scratches, it could be noticed that the scratch is surrounded by a pile of polymer that is higher in thickness compared to areas away from scratch boundaries. This suggests that the removed material during abrasion (or part of it) is being pushed into a different area rather than completely detached from the coating surface. This further supports that cohesion failure was not common, given that the abrasion force was, in some cases, enough to break the adhesion but not to completely overcome cohesion forces.

### 3.3 Influence of Particles

The effect of changing particle size was also investigated. Silica particles were chosen in this study and two different sizes were incorporated: Ø ∼ 15 nm and Ø ∼ 1.5 μm. In this part, the polymer used was PDMS(186) and was kept the same across all sample runs.

Previous reports using PDMS(184) have provided the determination of the optimum polymer/silica ratio for nSiO_2_, reported being 1.4.^29^ This same *M*_ratio_ was applied with PDMD(186) and was found to be suitable. To incorporate the μSiO_2_ similarly, *M*_ratio_ was investigated and optimized, and it was found that 0.3 was provided to maximize the superhydrophobicity of the coatings.

PDMS/nSiO_2_ and PDMS/μSiO_2_ coatings were prepared. Due to the difference in the optimum *M*_ratio_ between both particles, it was chosen to keep the polymer mass the same and change the silica quantity accordingly, as the polymer is expected to contribute largely to the coating robustness. The μSiO_2_ coating appeared to produce less coverage of the substrate; hence, the coating volume was needed to be doubled to ensure complete coverage. The images from the abrasion experiment are shown in Figure SI11a. The main observation was that the particle size influences the size of scratches made on the coatings. While the scratches in nSiO_2_ coatings tend to be narrow, μSiO_2_ coatings are prone to much wider scratches and material removal upon abrasion. If the coating is formed as aggregates of particles glued together by the polymer, and with the μSiO_2_ being 100 times larger in diameter, it is expected that these aggregates would cover bigger areas of the substrate. This causes the variation in consequences of the failure induced by the sandpaper, although this failure induction might occur similarly for both coatings. Comparing the results of image analysis, mass tracking, and mass difference suggests a similar conclusion, with the nanoparticles being more resistant to coating removal due to the smaller aggregates removed with each abrasion cycle (Figure SI11b).

**Figure 5:**
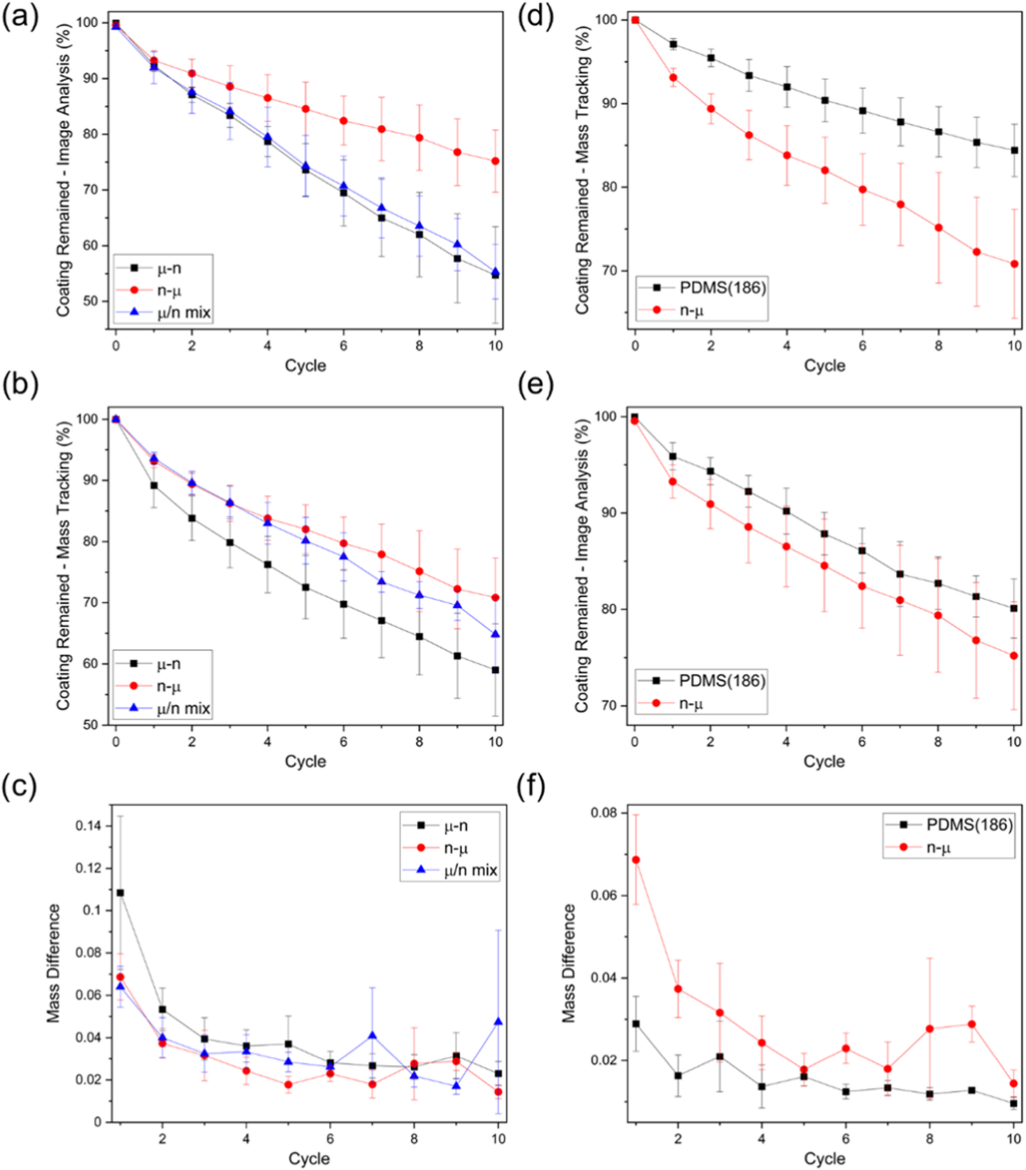
Plots for silica sample runs resilience performance vs. abrasion cycles (a–c) and a comparison between PDMS Sylgard 186 and n−μ resilience performance (d–f). (a, d) Percentage of coating remained as predicted by image analysis, (b, e) percentage of coating remained as measured by mass tracking, and (c, f) mass difference between every two consecutive cycles (normalized by dividing by the initial coating mass).

Another factor examined was the effect of combining different particle sizes in the coating. This would induce structural hierarchy, which is widely reported to enhance superhydrophobicity, but its effect on robustness is not as established. This inclusion was made in three different methods (as detailed in Section 2.4), either by spraying a layer of each particle size separately or by mixing both particles before the spraying process. The images from the abrasion experiment, along with the corresponding binary images and the estimated percentage of coating that remained, are shown in Figure SI12a–c for μ-n coatings, in Figure SI12d–f for n−μ coatings, and in Figure SI12g–i for n/μ mix coatings. Plots for coatings performance are shown in Figure 5a–c. It was observed that the least resistant was the μ–n coatings. They showed the highest coating removal percentage, as well as a faster degradation pattern in the first abrasion cycles as indicated by the mass difference plot. This is expected in light of the behavior of the PDMS/μSiO_2_ coatings explained above. While here there was an additional layer of smaller particles deposited, it appeared that the low adhesion of the micron particle aggregates forming the base of the coating influenced the coating robustness. A similar observation was noticed for the n−μ coatings, where the narrow, short scratches and less material removal resemble the abraded PDMS/nSiO_2_ coatings. While these combinations behaved predictably according to the base coating, comparing these coatings to each other (Figure 5d–f), the n−μ coatings appeared to show lower resilience. This suggests that the mixing of different particle sizes could introduce some irregularity that results in coatings more prone to failure.

The lower substrate adhesion shown by the μSiO_2_ can also explain how the n/μ mix coatings showed better resistance compared to μ–n coatings, as the former coatings would hypothetically have less of these particles at the base (caused by the particle mixing and the one-step spraying). Although this mixing would maximize the irregularity, making the coatings lose more mass toward the final abrasion cycles. This also agrees with the data shown in Table 1. While both (μ–n) and (n/μ mix) coatings show the same trend of incomplete coating detachment and high mass remaining than expected by image analysis, this was reversed for the n−μ coatings. This, again, is probably due to the low adhesion of the μSiO_2_ that occupies larger areas at the surface and can easily detach even if the nSiO_2_ particles are not removed.

The degradation of these composite materials is an extraordinarily complex process, as it involves consideration of coating cohesion and its adhesion to the underlying substrate. The cohesion of the composite material also includes polymer–nanoparticle interactions, in addition to the respective strengths of each component. Therefore, a full understanding of the degradation processes would only be achieved via a more comprehensive study. Another factor that is likely to play a role is the relative integration of particles within the polymer matrix (i.e., the homogeneity of the nanocomposite). SEM images of the nanocomposites (Figure SI13) show the morphology of the materials before and after abrasion. These are highly complex structures, with a range of morphological environments, i.e., variation in polymer thicknesses and nanoparticle densities/arrangements. This, as a result, provides a range of physical degradation pathways, and therefore differing localized resilience depending on the surrounding micro/nanostructure of the composite. This is simplified by using the image analysis method detailed within, as it does not give detailed localized degradation data. Instead, this approachable technique is able to provide an insight into degradation pathways, through examination of degradation imaging and mass-loss data.

## Conclusions

Resilience analysis of advanced materials and coatings is key in assessing their potential for real-world application. The methodology utilized here is demonstrated to provide insight into the fundamental resilience of various coating formulations.^28^ Enabling the identification of polymers and surface architectures that can provide maximized resistance to physical degradation (using the detailed abrasion technique). It was found that the resilience was maximized in PDMS coatings when PDMS(186) was incorporated, while selecting a higher-*M*_w_ polymer for PVC coatings enhanced the performance. In addition, nano-sized silica performed better than the micron-sized particles, suggesting slower degradation patterns for coatings involving smaller particles. These resilience data were used to quantitatively compare different coatings, and due to the two measures used (image analysis and mass difference), an insight into respective degradation pathways can be gained. This is highlighted in the comparison between PDMS and PVC coatings, whereby PVC coatings provide a substantially higher difference between these two measures, with a higher degradation detected through sample weighing. In addition, the nature of scratches was investigated via confocal fluorescence microscopy, where PDMS coatings material showed a noncomplete detachment from the surface once being removed via abrasion.

The methodology is fully reproducible, with full details of the procedure and the code required provided in a previous publication.^28^ The study of nanocomposites is extremely challenging, as nano/microstructures can vary locally, which is dependent on a range of factors (including the relative integrations of formulation components).^38^ The reported methodology and results presented herein demonstrate clear applicability to the study of their resilience, with advanced techniques (e.g., confocal fluorescence imaging) able to provide a more in-depth analysis.

The work presented demonstrates the potential for wider implementation both in academic and commercial sectors. The quantification of materials degradation is a complicated principle, which becomes even more challenging when there is neither a standardized degradation nor analysis protocol. This is a particular challenge in the development of physically robust superhydrophobic materials, whereby relative resilience is generally low, and there is a tremendous divergence in the testing methodologies employed. The reported methodology provides the ability to perform a standardized analysis while enabling flexibility in the degradation techniques used. If widely adopted, this would allow for the quantitative comparison of materials, and more straightforward development of more robust materials.

## Supporting Information Available

Adjustment of *M*_ratio_ for PDMS/SiO_2_ coatings; the utilized abrasion analysis method; dimensions for dog-bone metal cutter and the utilized dog-bone mold; method for preparing PVC films; mechanical properties of PVC; full image sets for PVC-based, PDMS-based, and silica-based coatings; SEM images of PDMS coatings; noise correction for PDMS coatings; and stress–strain curves for PVC and PDMS (PDF)
