# Supplementary material for: Study on the Influence of Polymer/Particle Properties on the Resilience of Superhydrophobic Coatings
Source: ACS Omega. 2022 May 18;7(21):18052–62. doi: 10.1021/acsomega.2c01547 (PMC9161389; doi:10.1021/acsomega.2c01547)
Supplement: Supplementary file 1 — ao2c01547_si_001.pdf [file ao2c01547_si_001.pdf]

A Study on the Influence of Polymer/Particle Properties on the Resilience of Superhydrophobic Coatings

Yasmin A. Mehanna[1,2], Colin R. Crick[2]*

[1] Materials Innovation Factory, Department of Chemistry, University of Liverpool, Liverpool, L69 7ZD, UK

[2] School of Engineering and Materials Science, Queen Mary University of London, Mile End Road, London E1 4NS, UK

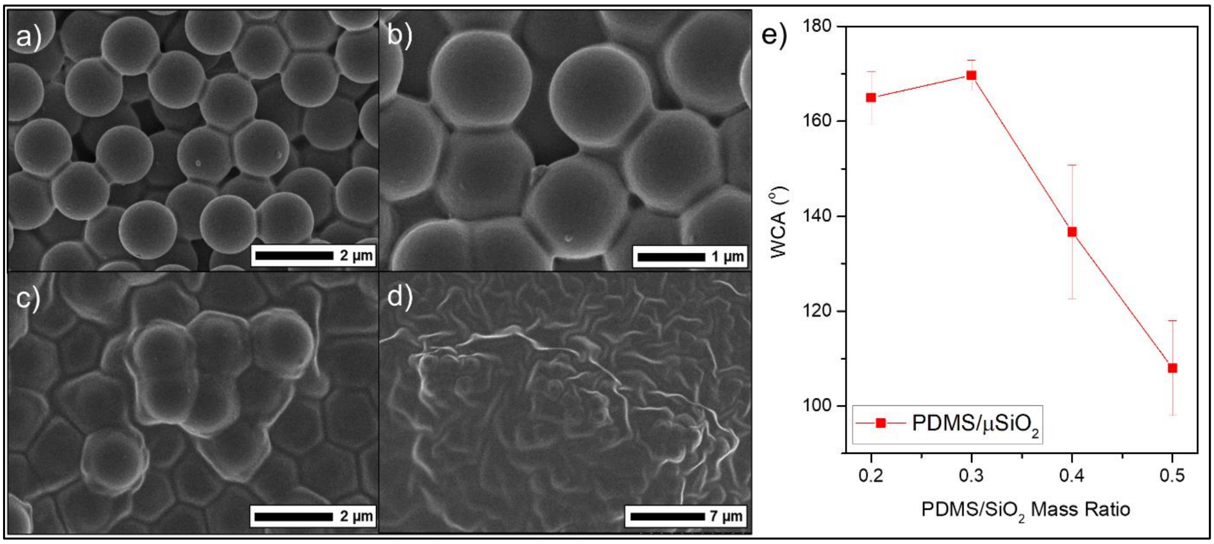

Figure SI1: a-d) SEM images of PDMS/μSiO_2 coatings prepared using different polymer/silica mass ratios. The typical mass ratios shown are a) 0.2, b) 0.3, c) 0.4 and d) 0.5. Scale bars are shown for each image. e) Plot of the WCAs measured for these coatings.

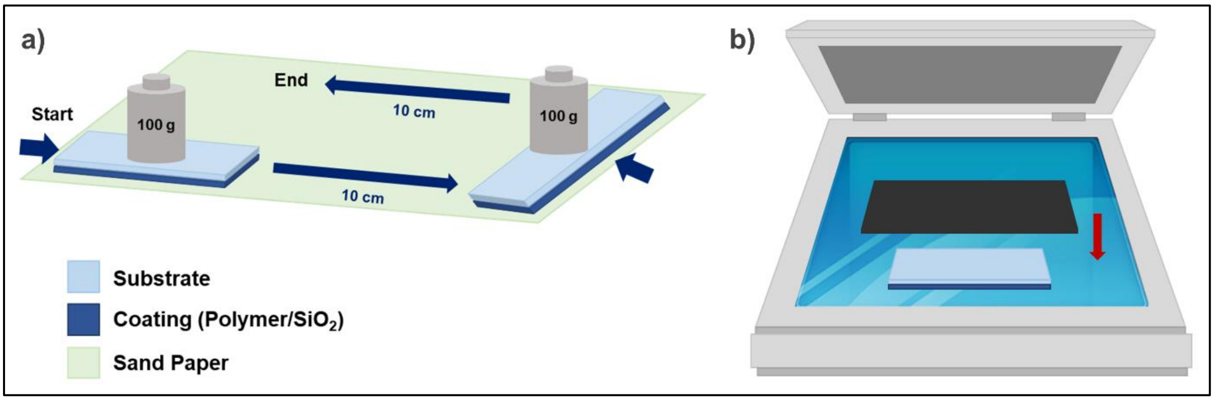

Figure SI2: The abrasion analysis method, involved abrasion cycles (a) and scanning (b). a) The coated substrate was placed face-down onto sandpaper (grit no. 120) with a 100 g-weight placed on top of it. Both the substrate and weight were pushed for 10 cm, before being turned 90° and moved a further 10 cm to complete one cycle. b) The coated substrate was placed face-down with a black paper card on top of it to ensure a dark background for high contrast.

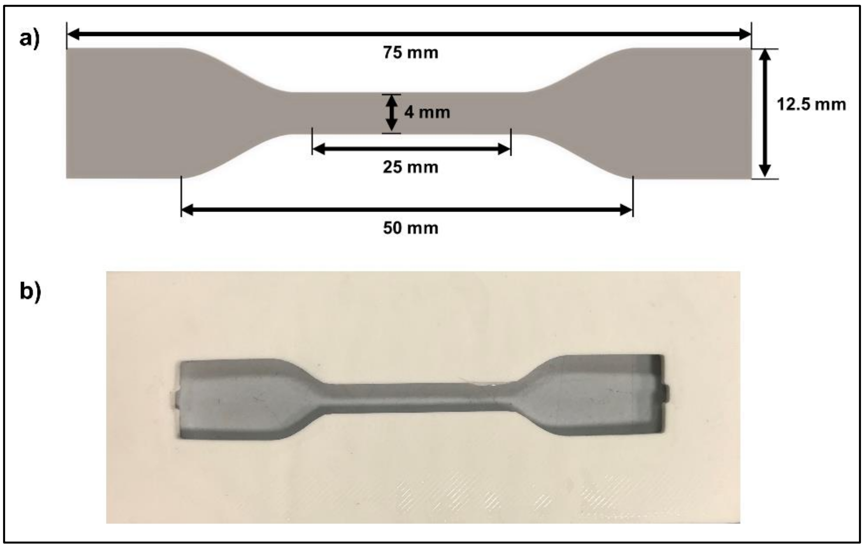

Figure SI3: a) A sketch of the dog-bone metal cutter used for PVC films with dimensions highlighted, and b) an image of the 3D-printed PLA mould used for PDMS samples.

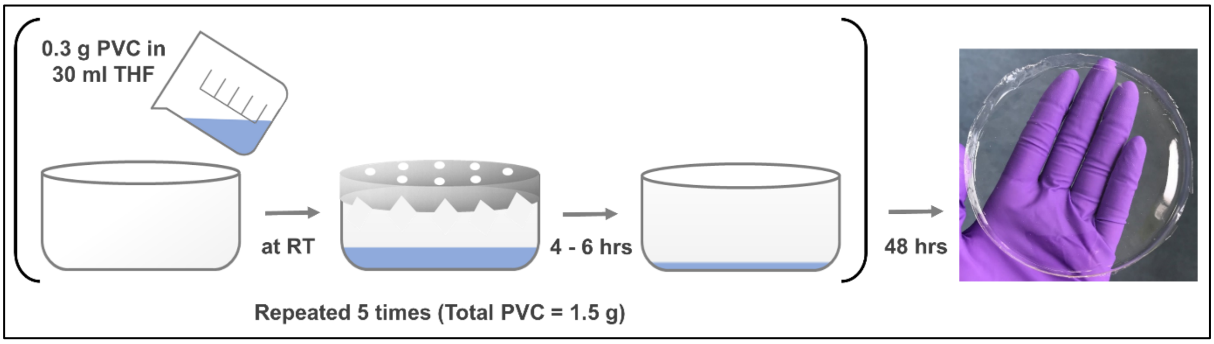

Figure SI4: Method for preparing PVC films for mechanical testing. PVC powder was solvated in THF and left to dry in a crystallization dish (Ø = 11.5 cm) covered with aluminium foil at room temperature for 4-6 hrs. This was repeated five times (total PVC quantity = 1.5). The film was removed after complete solvent evaporation (48 hrs from the last solution added).

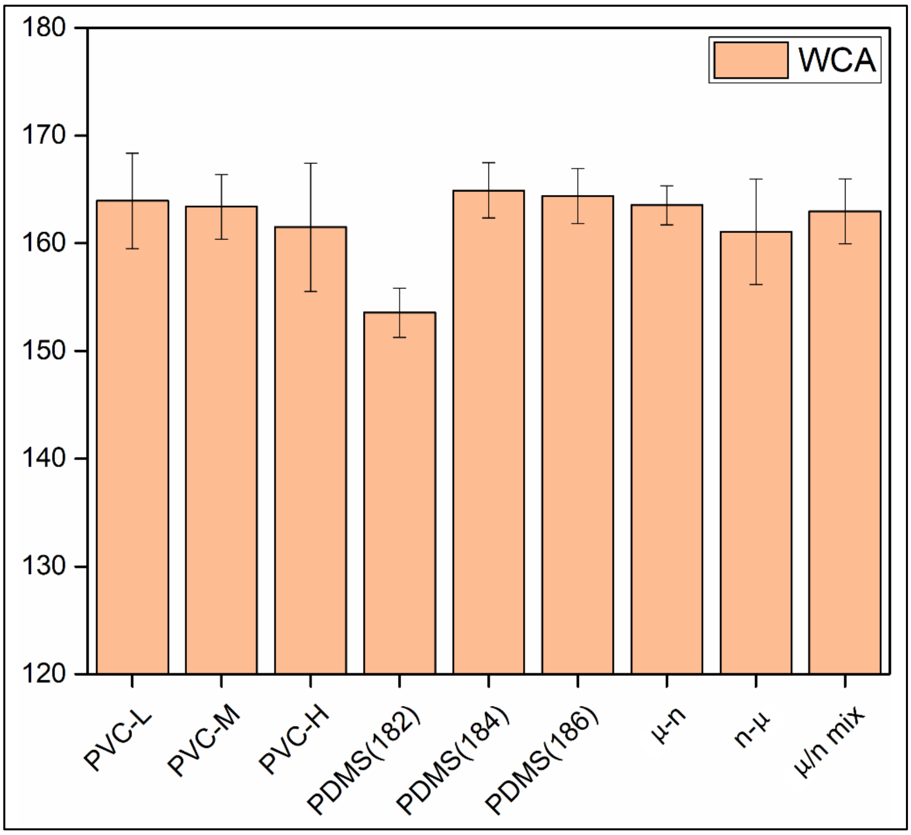

Figure SI5: WCAs for the coatings tested in this study, showing their superhydrophobicity before abrasion.

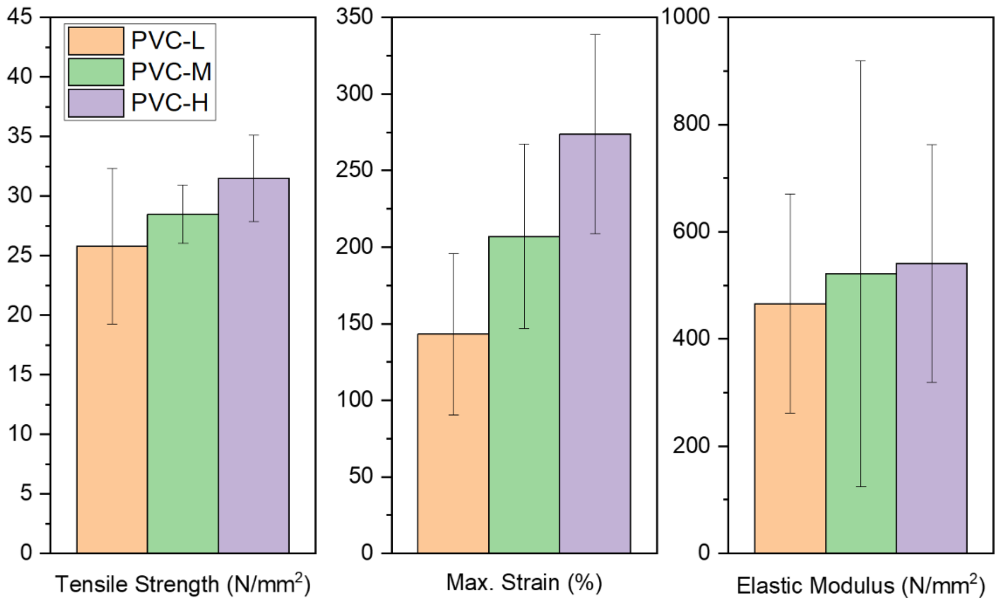

Figure SI6: Tensile strength, maximum strain, and elastic modulus results for PVC polymers.

Figure SI7: The full image sets for the sample runs of PVC with different molecular weights, showing the coloured (upper), binary (lower) images, and the associated percentage of coating remained as predicted by image analysis. (a-c) PVC-L runs, (d-f) PVC-M runs, (g-i) PVC-H runs.

a) PVC-L – Run No.1:

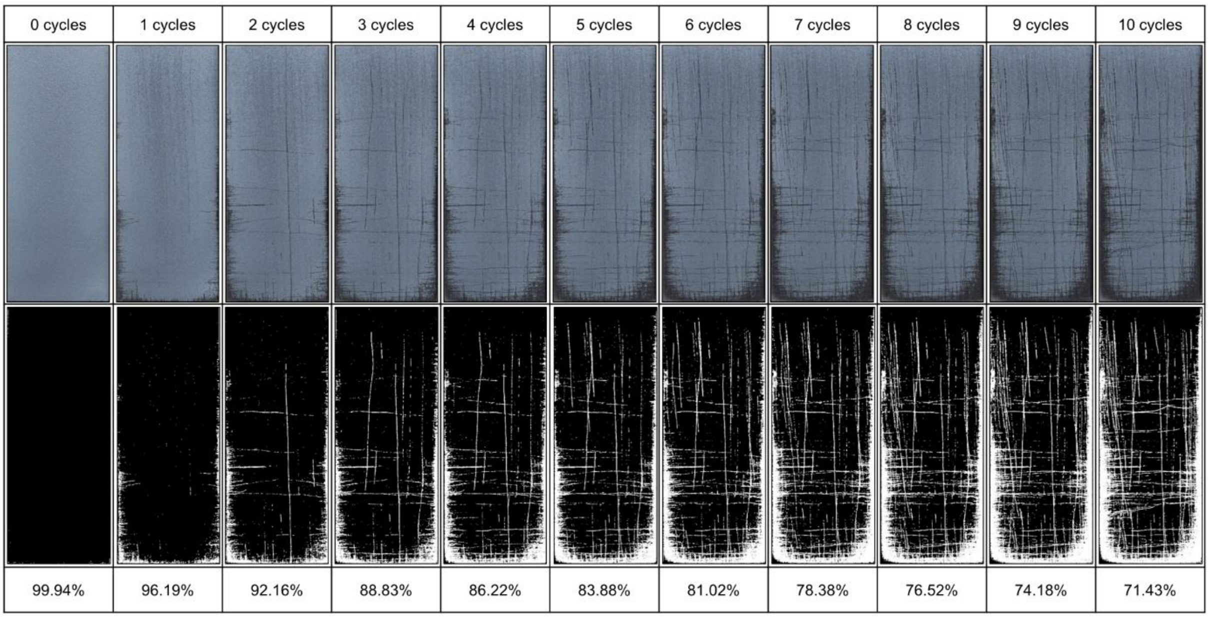

b) PVC-L – Run No.2:

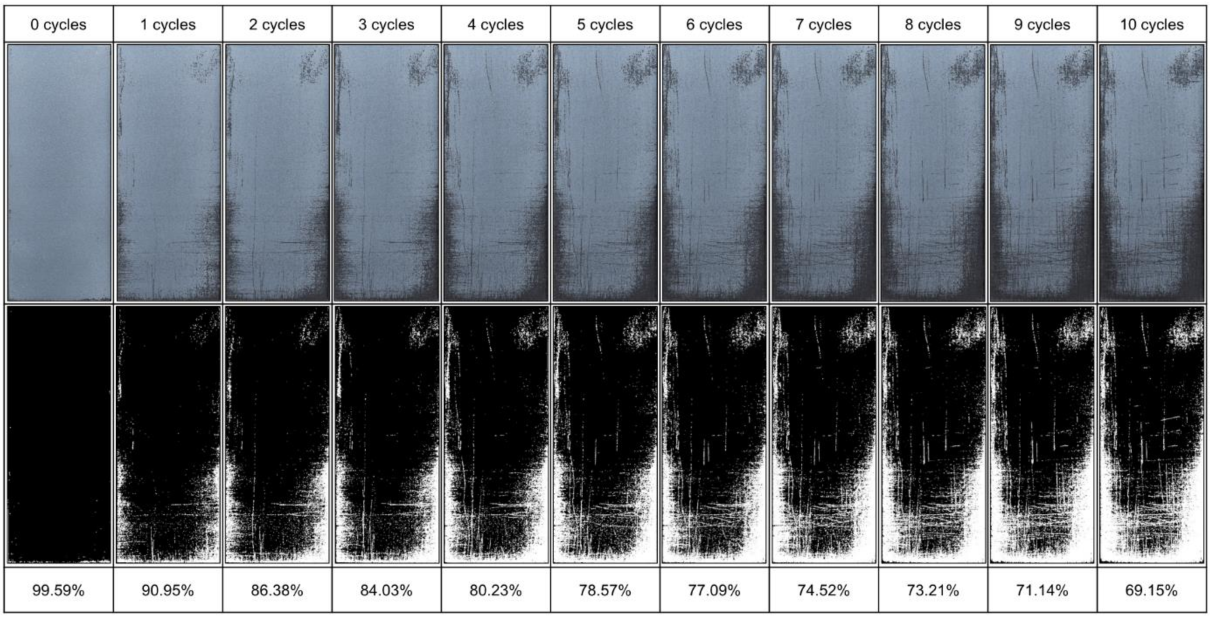

c) PVC-L – Run No.3:

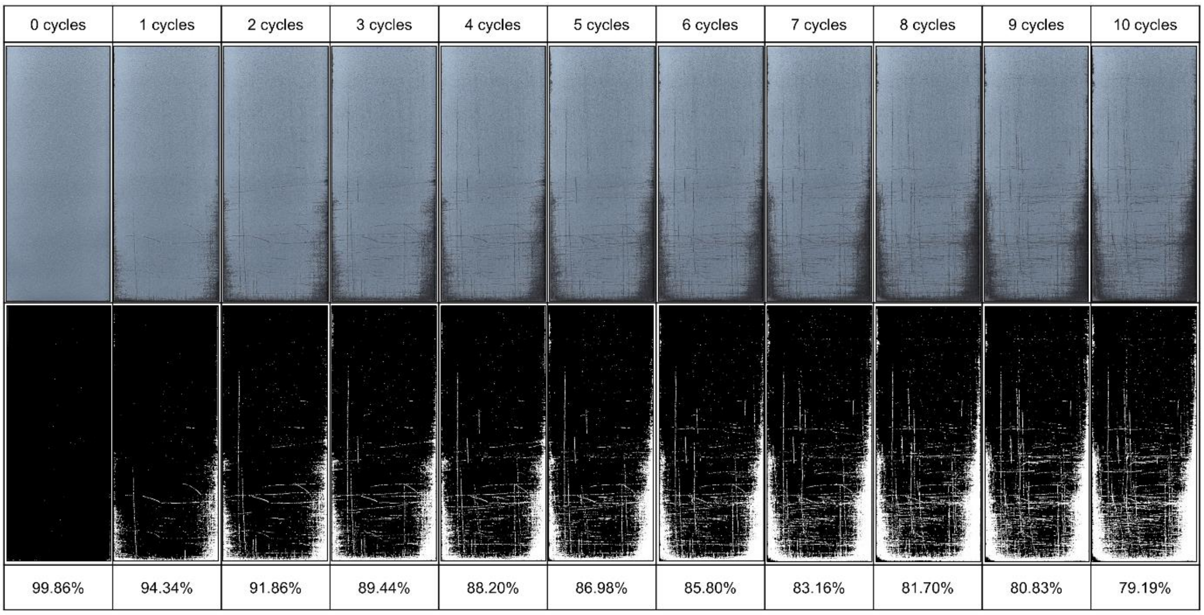

d) PVC-M – Run No.1:

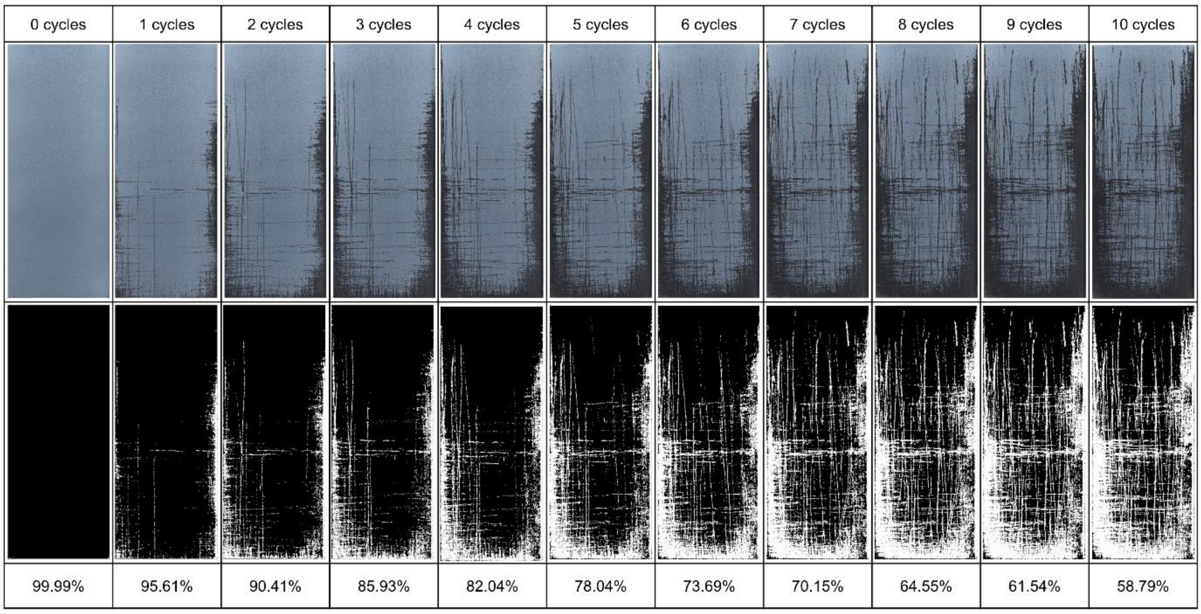

e) PVC-M – Run No.2:

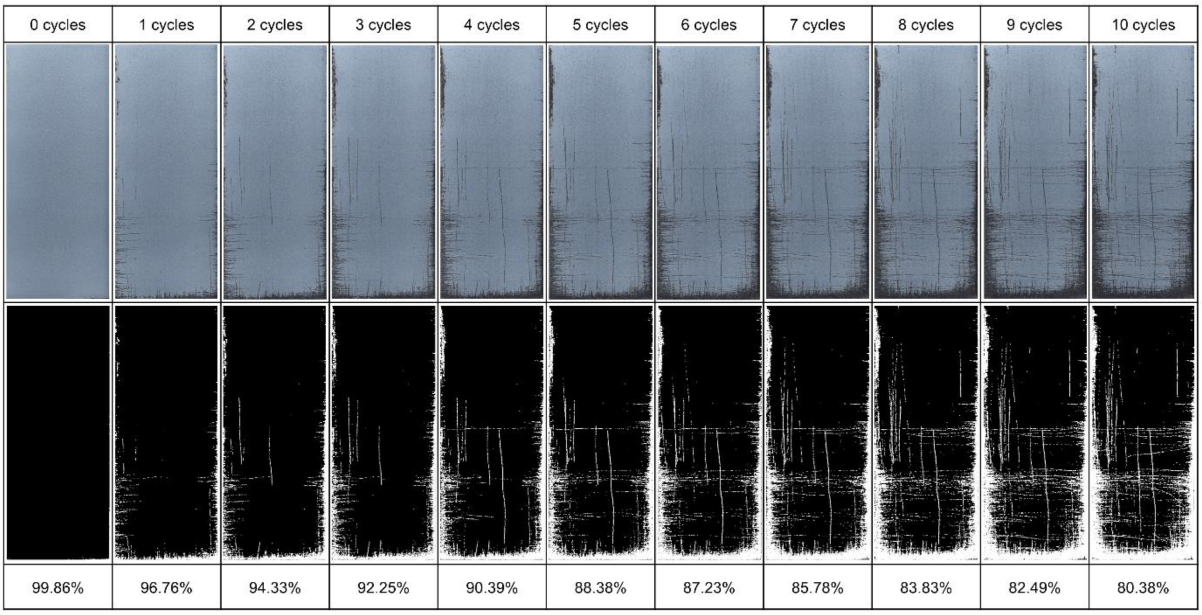

f) PVC-M – Run No.3:

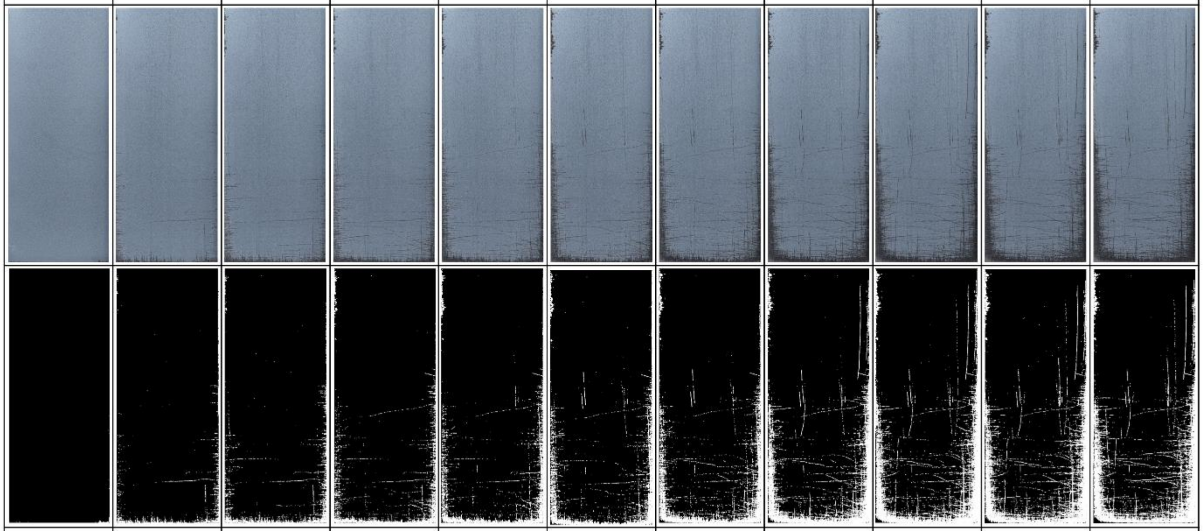

0 cycles	1 cycles	2 cycles	3 cycles	4 cycles	5 cycles	6 cycles	7 cycles	8 cycles	9 cycles	10 cycles
99.94%	98.23%	96.95%	95.77%	94.24%	93.19%	91.45%	88.51%	86.55%	85.95%	84.68%

g) PVC-H – Run No.1:

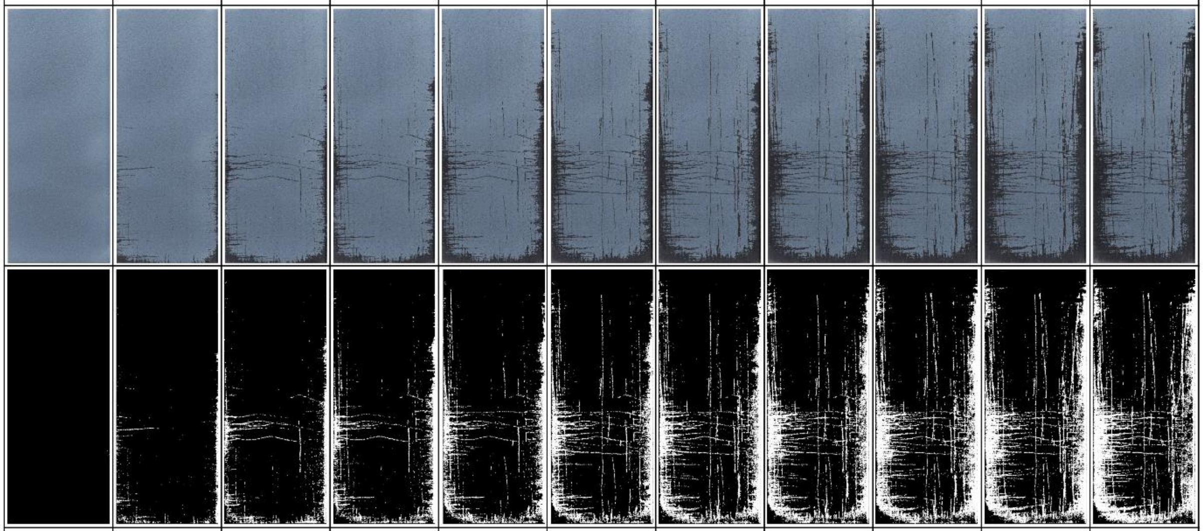

0 cycles	1 cycles	2 cycles	3 cycles	4 cycles	5 cycles	6 cycles	7 cycles	8 cycles	9 cycles	10 cycles
100.00%	98.01%	94.58%	91.90%	88.78%	85.45%	83.09%	80.93%	78.95%	76.79%	74.88%

h) PVC-H – Run No.2:

0 cycles	1 cycles	2 cycles	3 cycles	4 cycles	5 cycles	6 cycles	7 cycles	8 cycles	9 cycles	10 cycles
99.98%	97.96%	96.13%	94.58%	92.58%	90.13%	88.25%	86.14%	84.99%	83.08%	81.40%

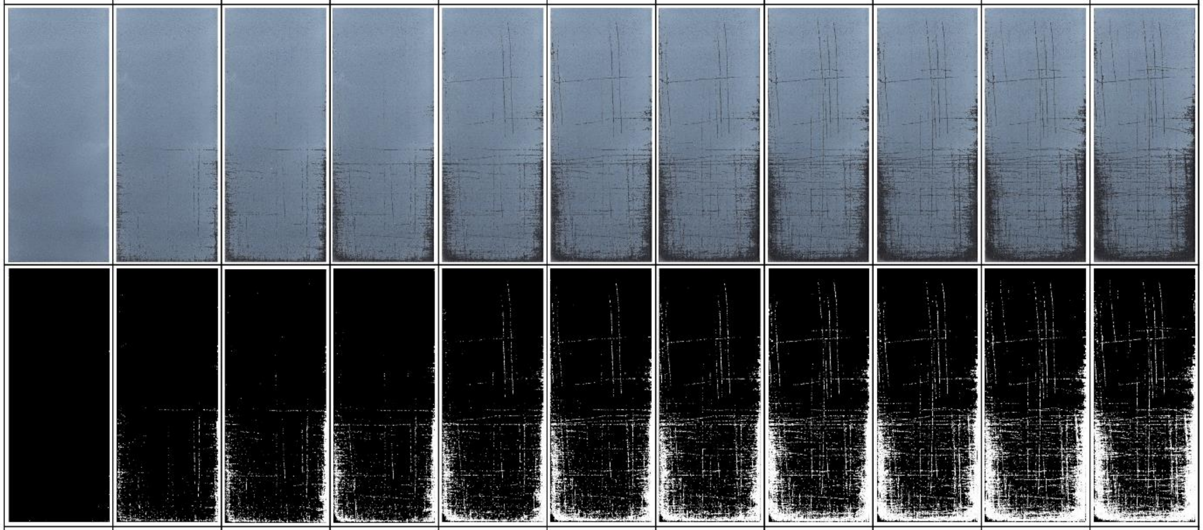

i) PVC-H – Run No.3:

0 cycles	1 cycles	2 cycles	3 cycles	4 cycles	5 cycles	6 cycles	7 cycles	8 cycles	9 cycles	10 cycles
99.96%	98.91%	97.90%	97.36%	96.62%	95.31%	94.44%	93.04%	92.10%	90.51%	89.86%

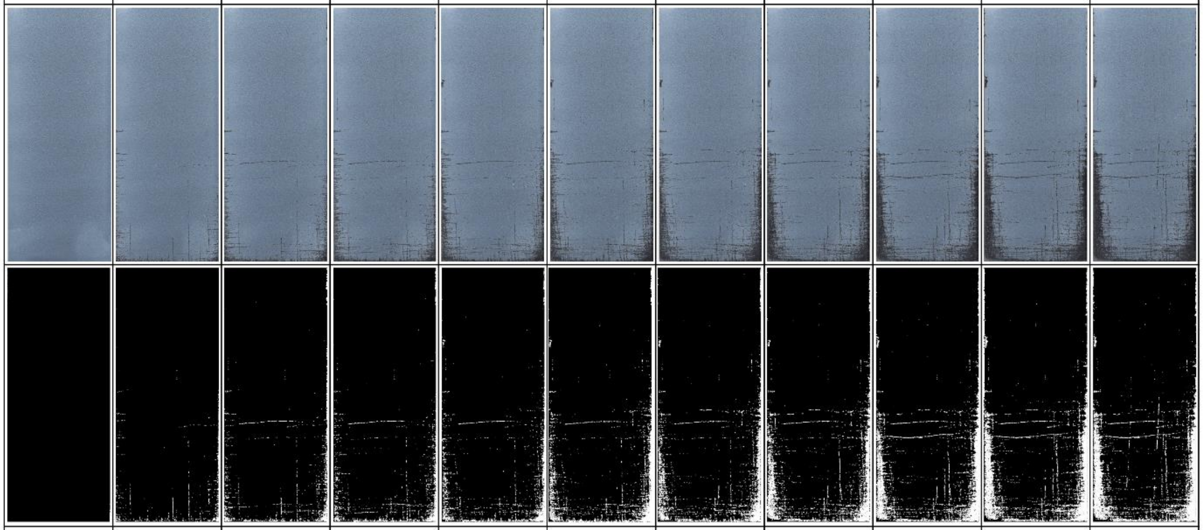

Figure SI8: The full image sets for the sample runs of PDMS with different tensile strengths, showing the coloured (upper), binary (lower) images, and the associated percentage of coating remained as predicted by image analysis. (a-c) PDMS Sylgard 182 runs, (d-f) PDMS Sylgard 184 runs, (g-i) PDMS Sylgard 186 runs.

a) PDMS (182) – Run No.1:

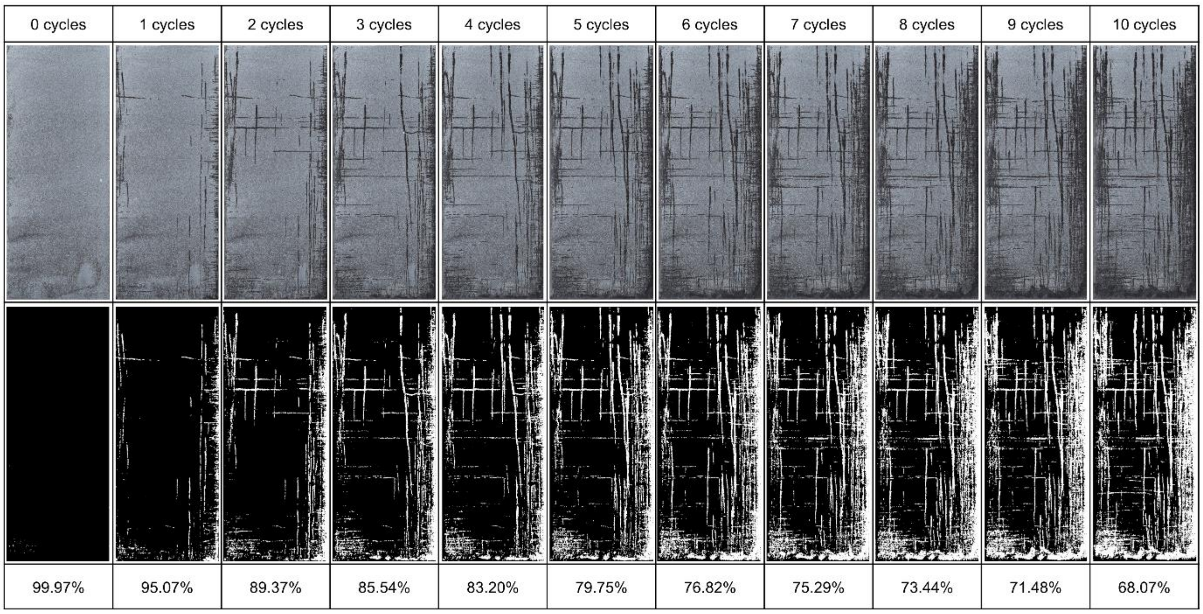

b) PDMS (182) – Run No.2:

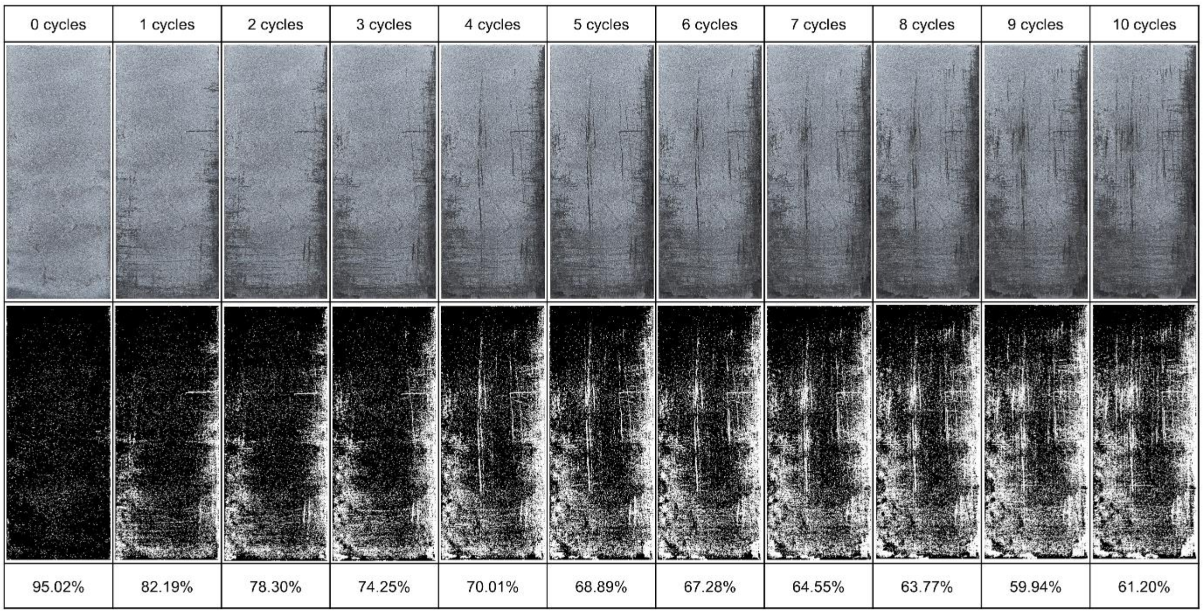

c) PDMS (182) – Run No.3:

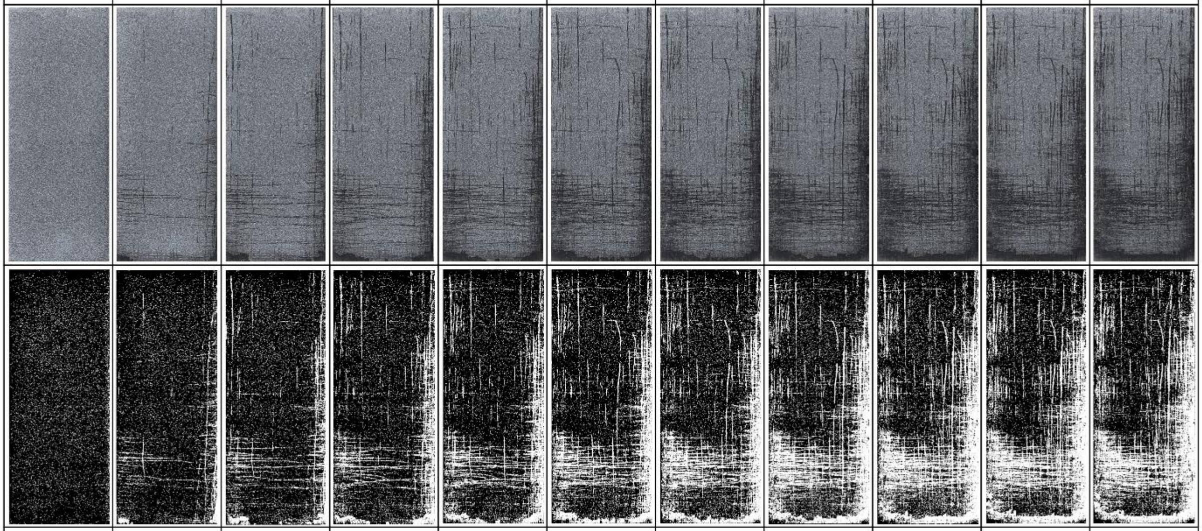

0 cycles	1 cycles	2 cycles	3 cycles	4 cycles	5 cycles	6 cycles	7 cycles	8 cycles	9 cycles	10 cycles
88.94%	85.73%	81.27%	77.96%	75.45%	71.39%	67.48%	63.18%	60.63%	61.10%	59.37%

d) PDMS (184) – Run No.1:

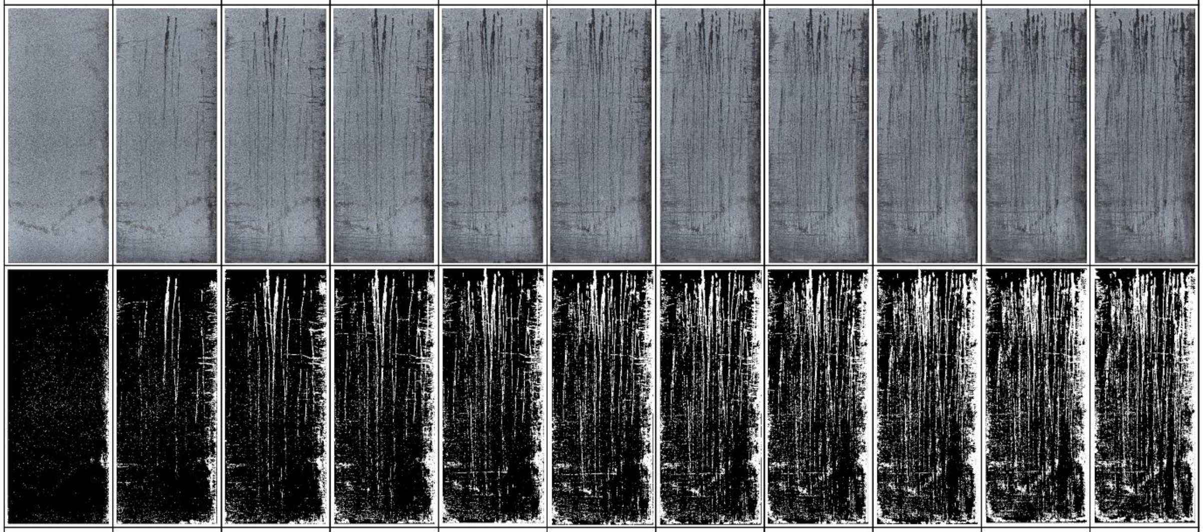

0 cycles	1 cycles	2 cycles	3 cycles	4 cycles	5 cycles	6 cycles	7 cycles	8 cycles	9 cycles	10 cycles
96.86%	91.36%	88.05%	84.05%	81.45%	78.72%	77.18%	76.06%	72.91%	71.89%	69.66%

e) PDMS (184) – Run No.2:

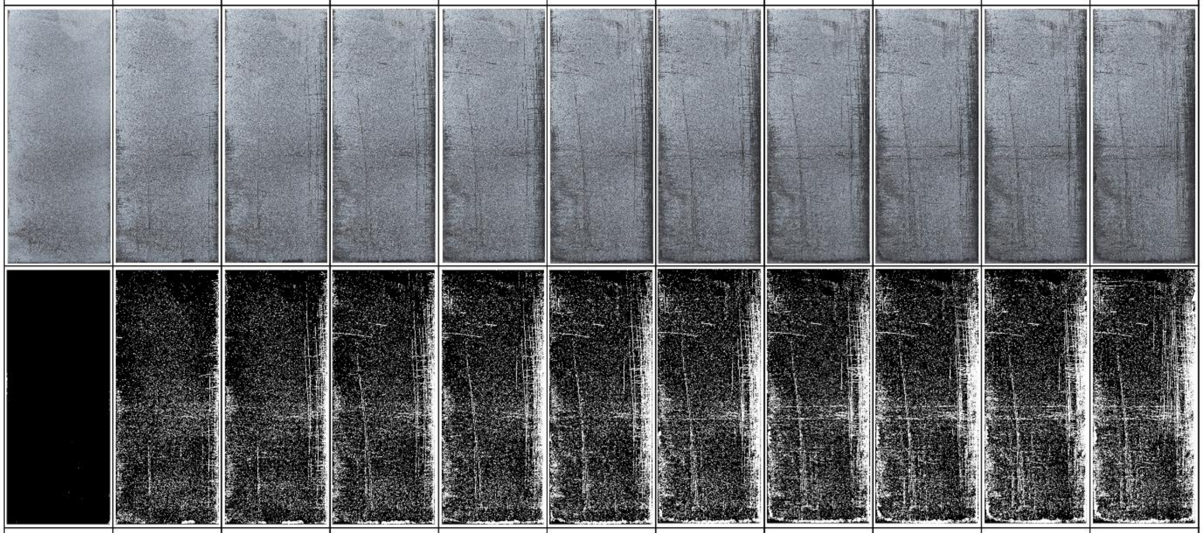

0 cycles	1 cycles	2 cycles	3 cycles	4 cycles	5 cycles	6 cycles	7 cycles	8 cycles	9 cycles	10 cycles
99.96%	82.28%	79.87%	78.21%	75.86%	74.79%	72.81%	72.20%	71.52%	71.48%	69.22%

f) PDMS (184) – Run No.3:

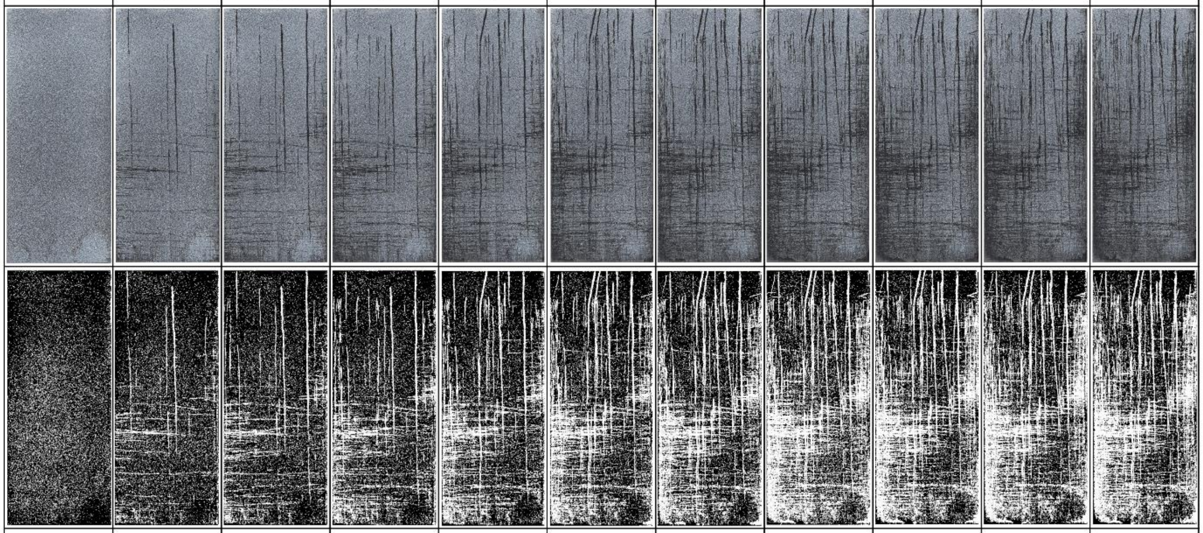

0 cycles	1 cycles	2 cycles	3 cycles	4 cycles	5 cycles	6 cycles	7 cycles	8 cycles	9 cycles	10 cycles
77.20%	76.44%	73.30%	68.69%	64.97%	60.22%	57.75%	50.38%	53.16%	51.61%	50.08%

g) PDMS (186) – Run No.1:

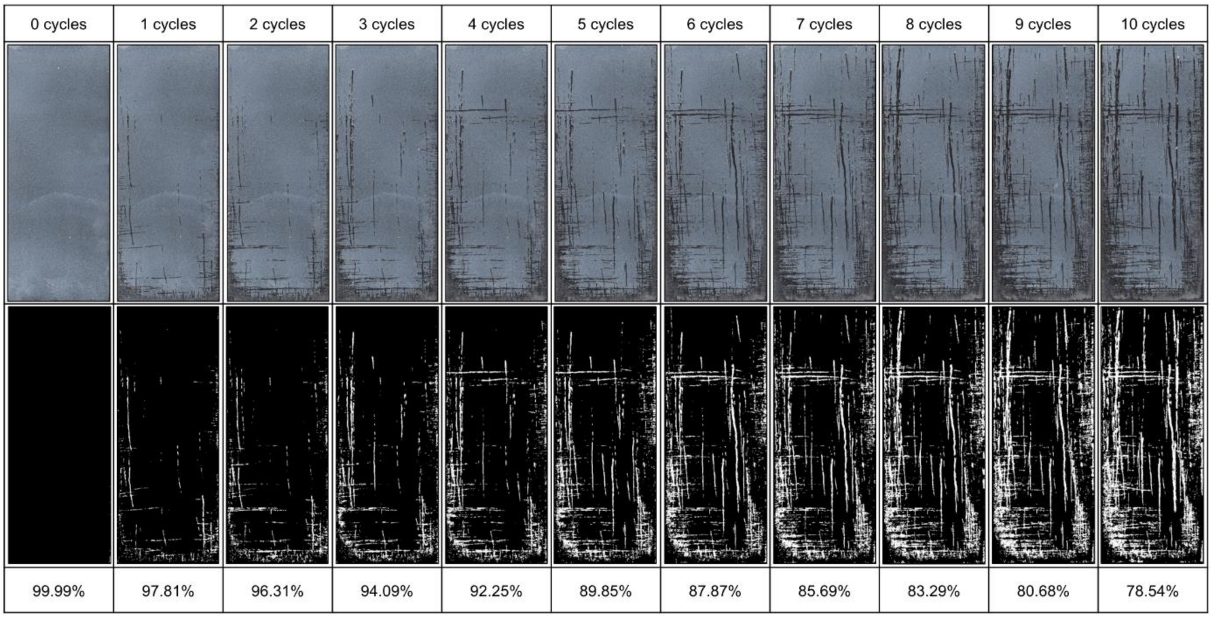

h) PDMS (186) – Run No.2:

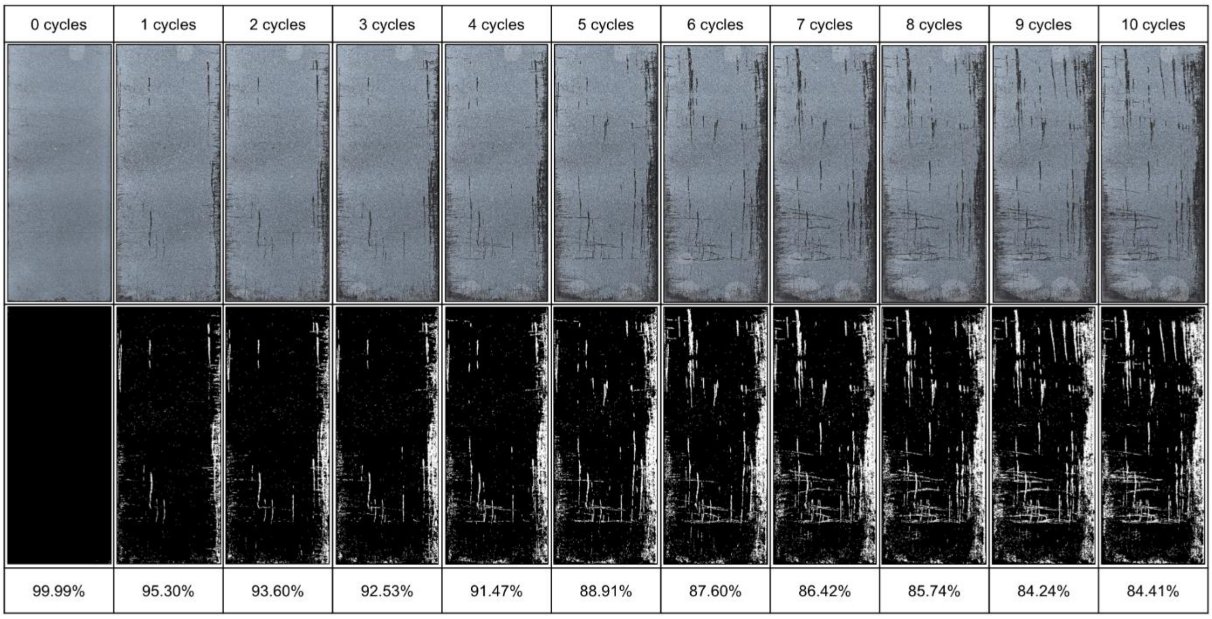

i) PDMS (186) – Run No.3:

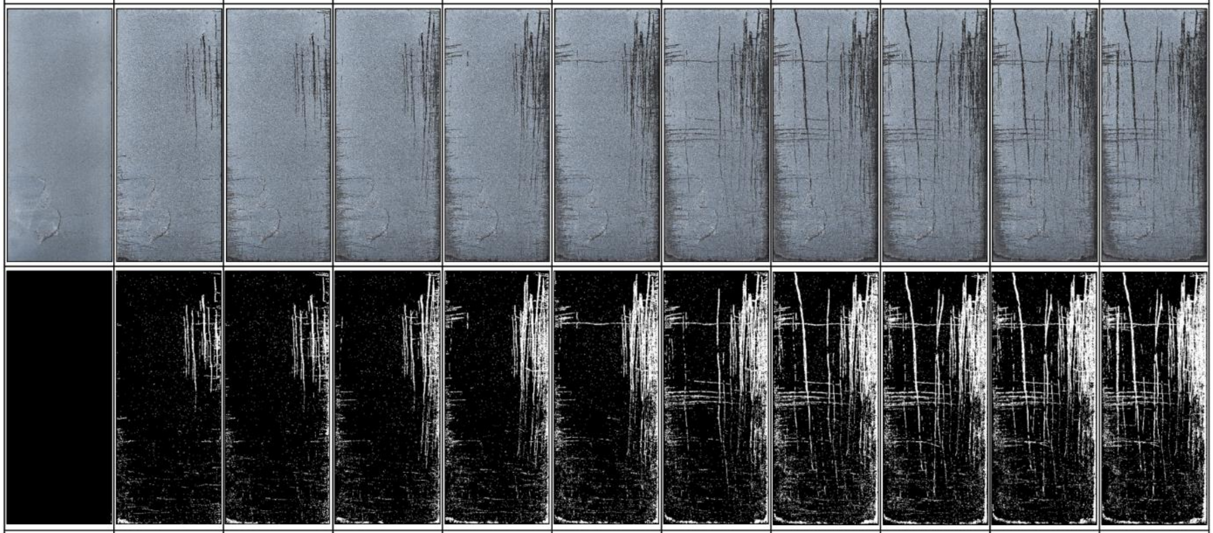

0 cycles	1 cycles	2 cycles	3 cycles	4 cycles	5 cycles	6 cycles	7 cycles	8 cycles	9 cycles	10 cycles
100.00%	94.51%	93.10%	90.10%	86.87%	84.80%	82.83%	78.93%	79.12%	79.13%	77.37%

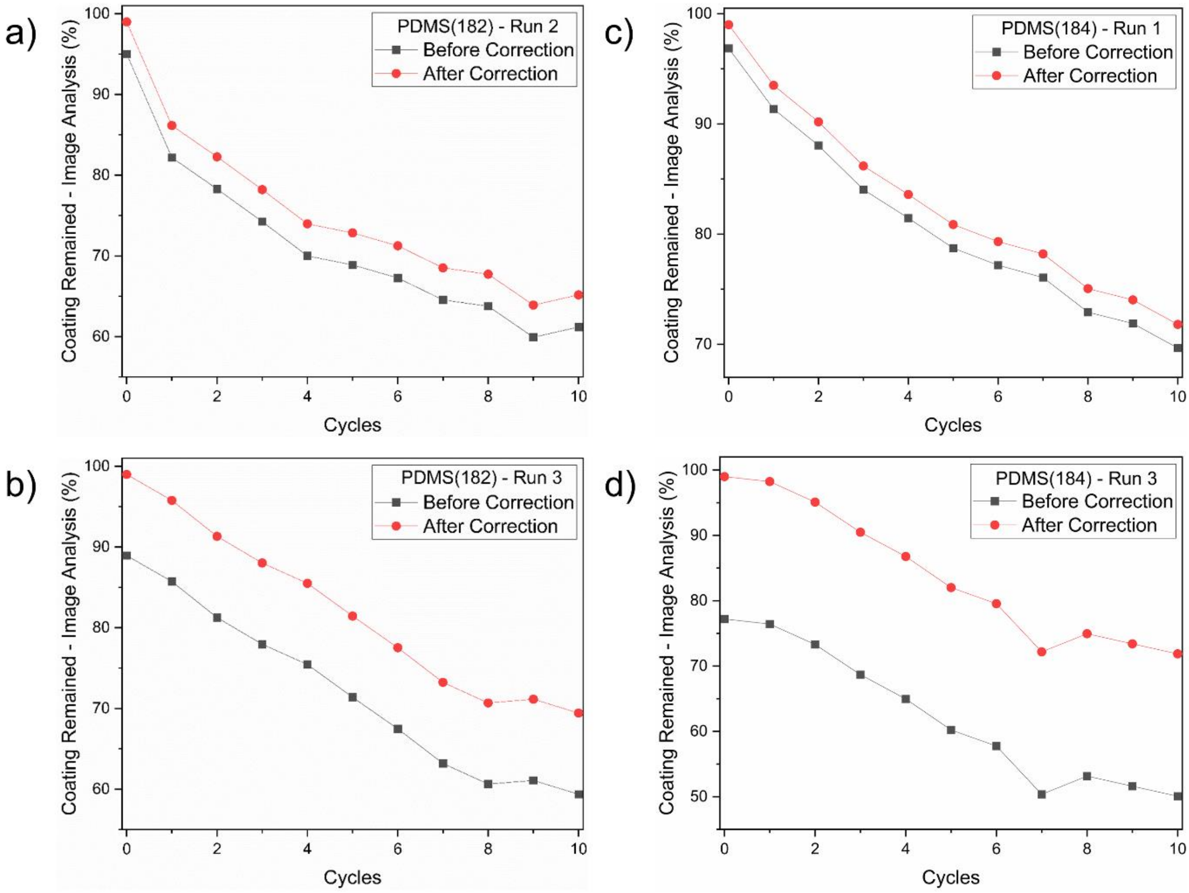

Figure SI9: Image analysis values before and after correction for PDMS(182) (Run 2 (a) and Run 3 (b)) and PDMS(184) (Run 1 (c) and Run 3 (d)).

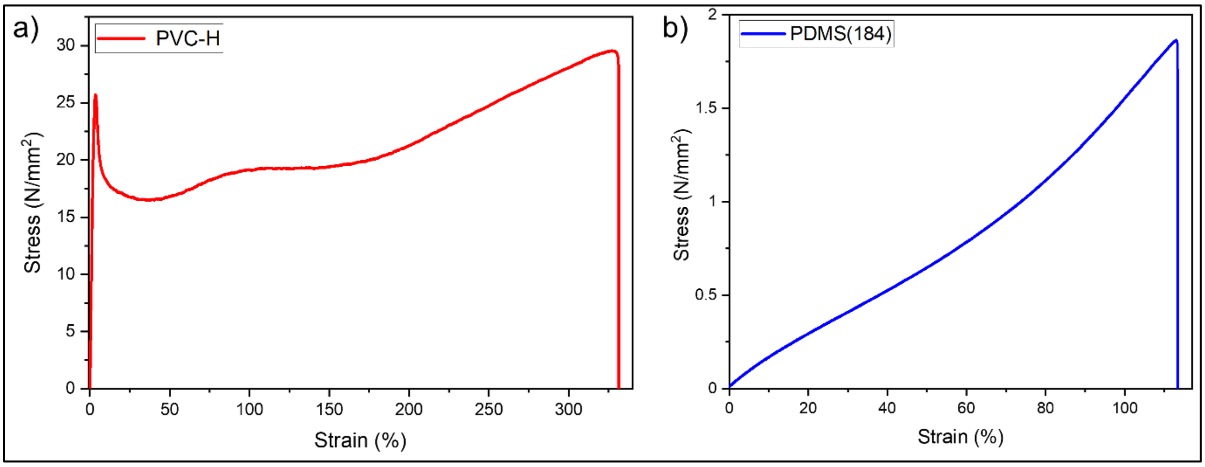

Figure SI10: Stress-strain curve for a) PVC and b) PDMS

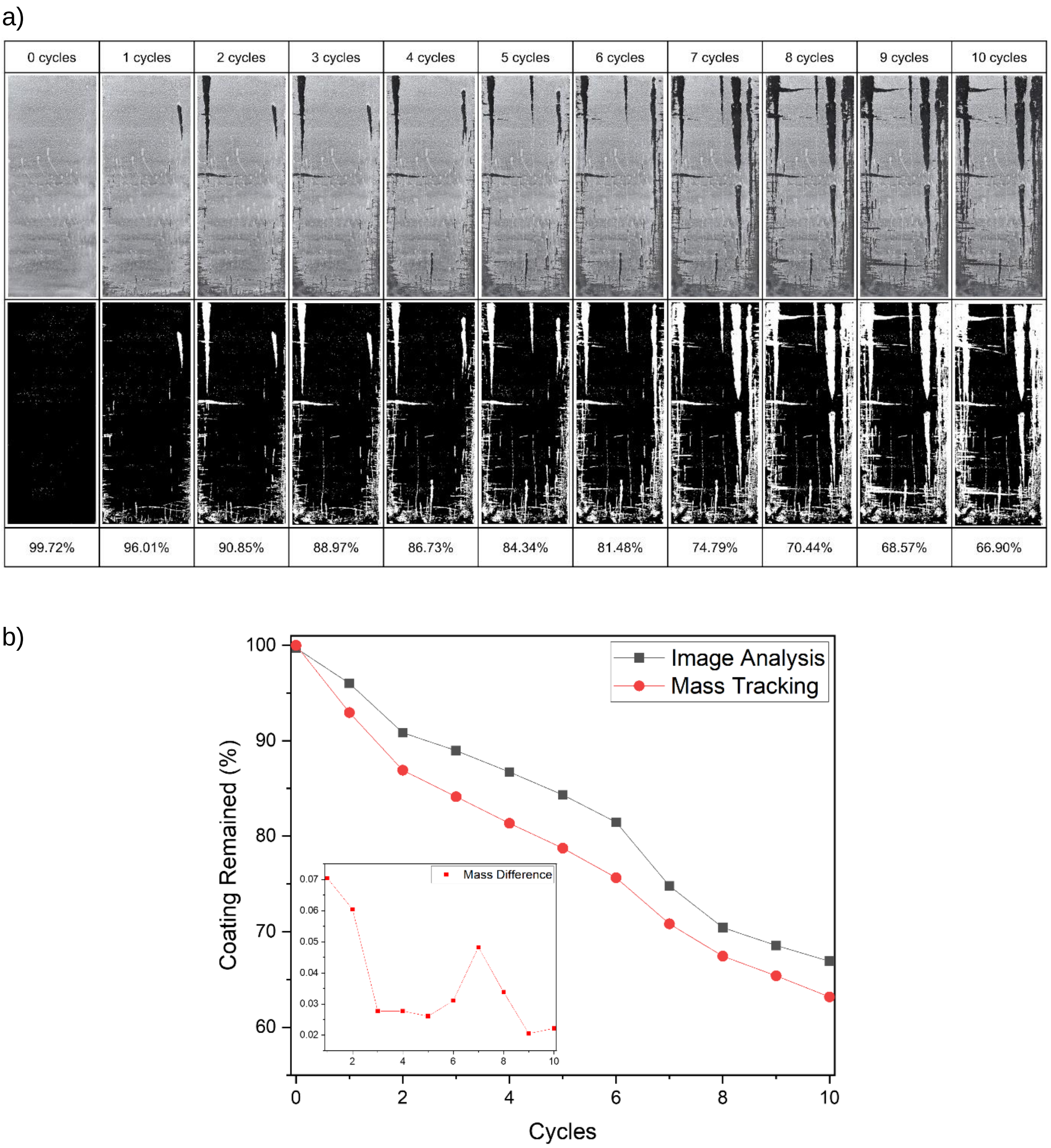

Figure SI11: a) The image set for the sample run of μSiO_2 coating, showing the coloured (upper), binary (lower) images, and the associated percentage of coating remained as predicted by image analysis. b) Plot for Image analysis, mass tracking and mass difference (inset) results of μSiO_2 coating.

Figure SI12: The full image sets for the sample runs of silica with different sizes and deposition order, showing the coloured (upper), binary (lower) images, and the associated percentage of coating remained as predicted by image analysis. (a-c) µ-n runs, (d-f) n-µ runs, (g-i) µ/n mix runs.

a) µ-n – Run No.1:

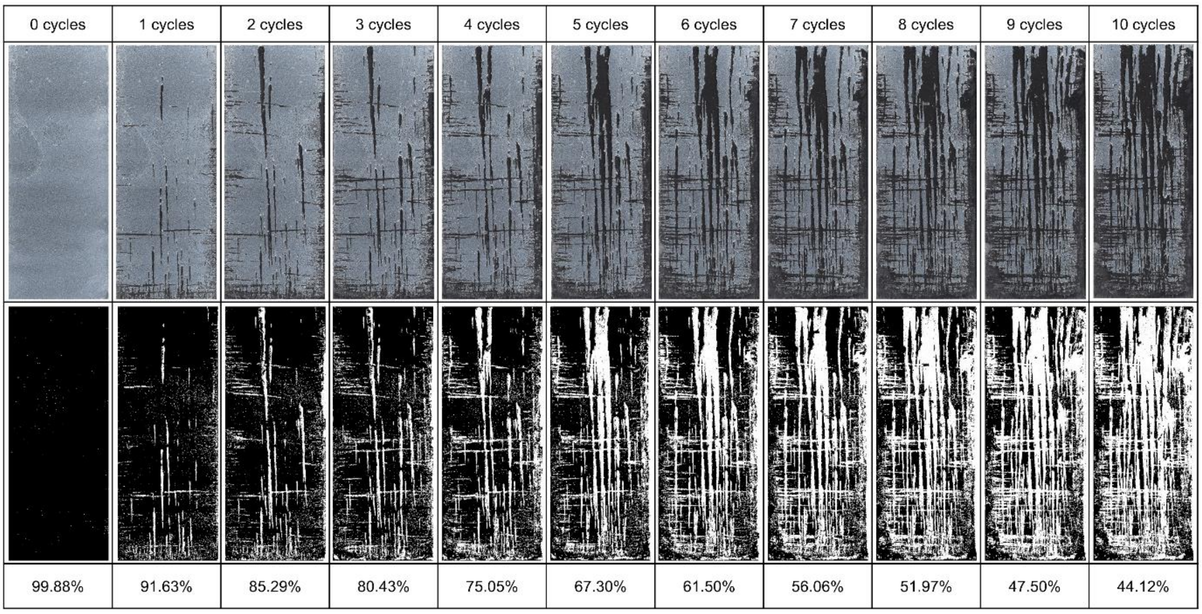

b) µ-n – Run No.2:

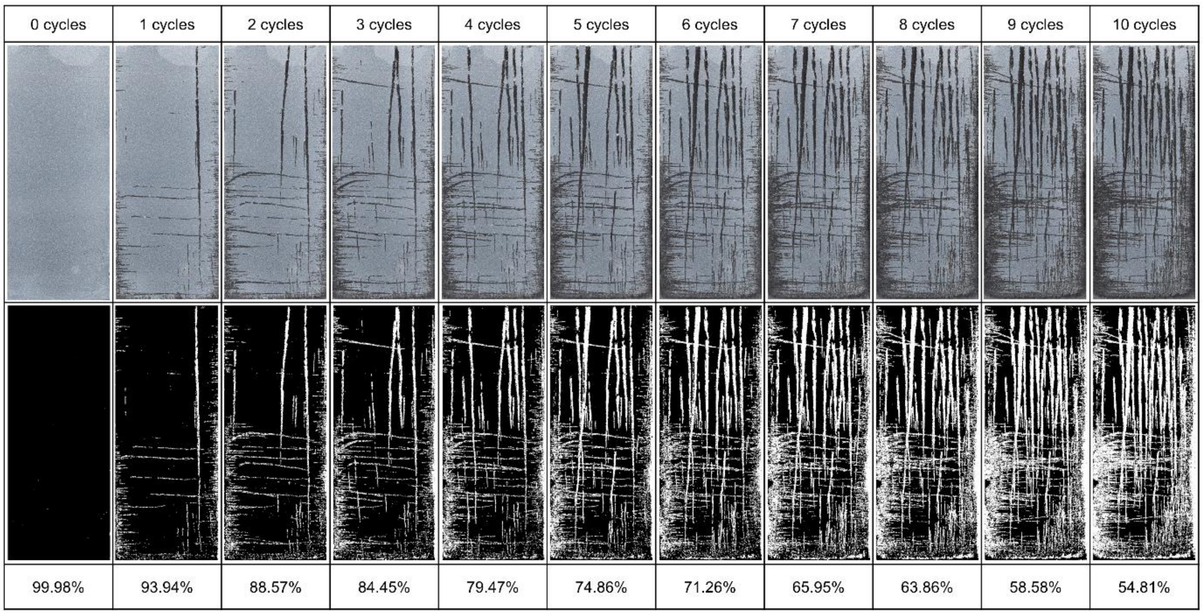

c) µ-n – Run No.3:

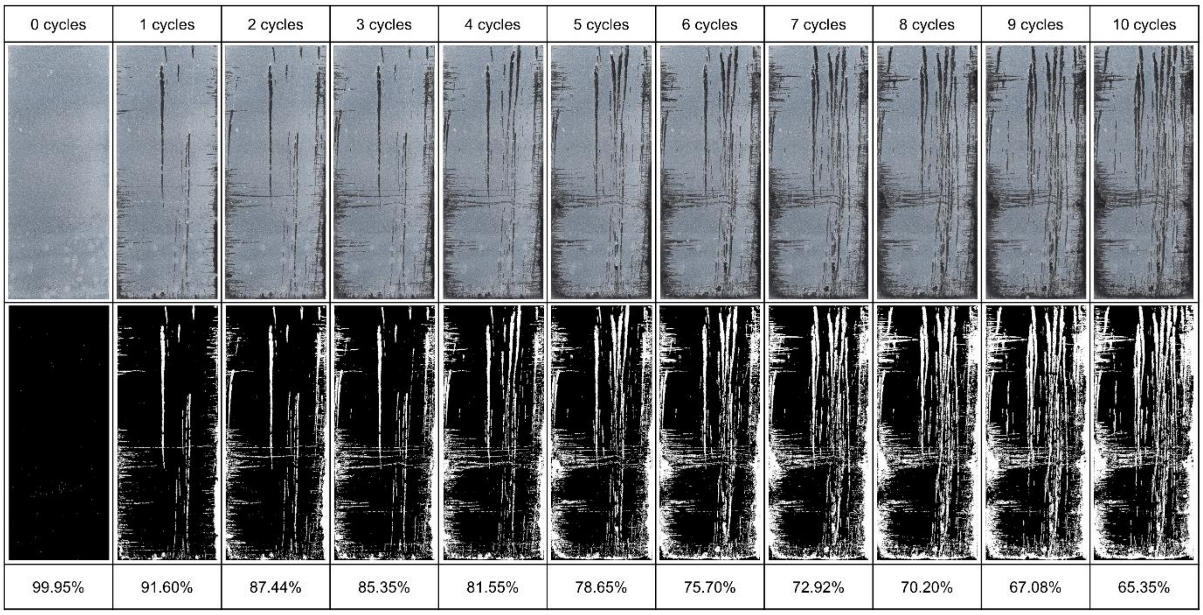

d) n-µ – Run No.1:

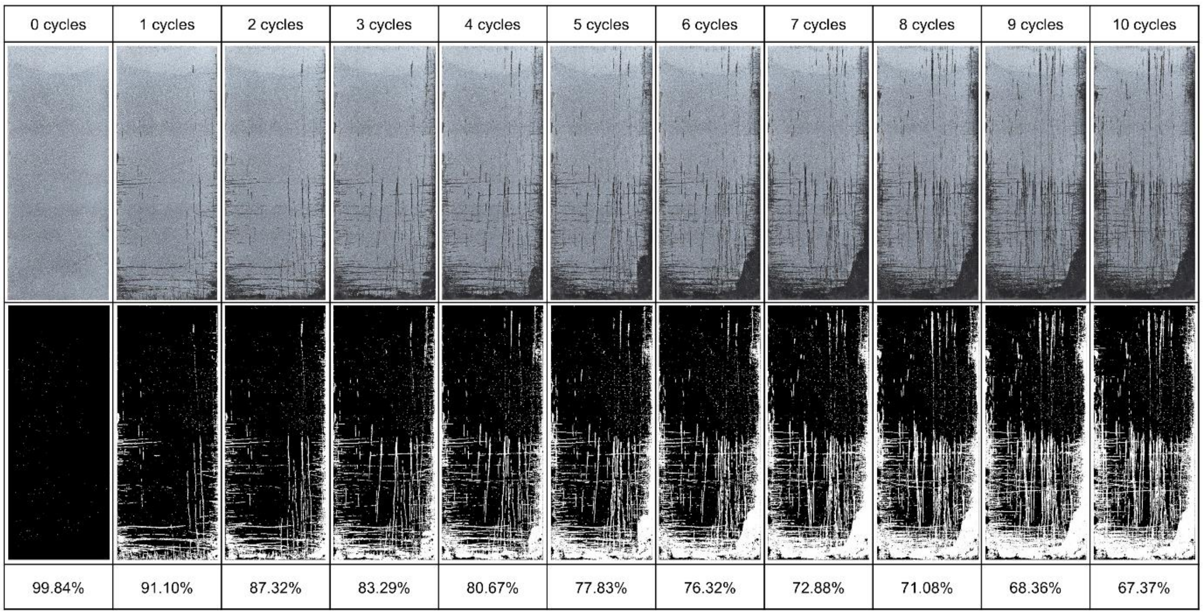

e) n-µ – Run No.2:

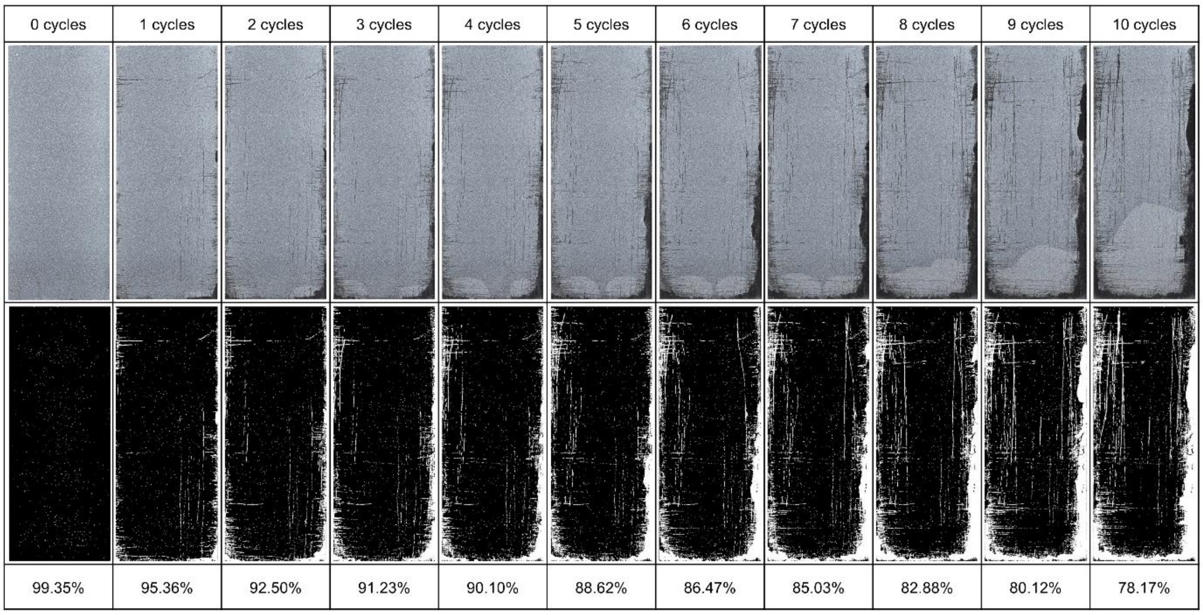

f) n-µ – Run No.3:

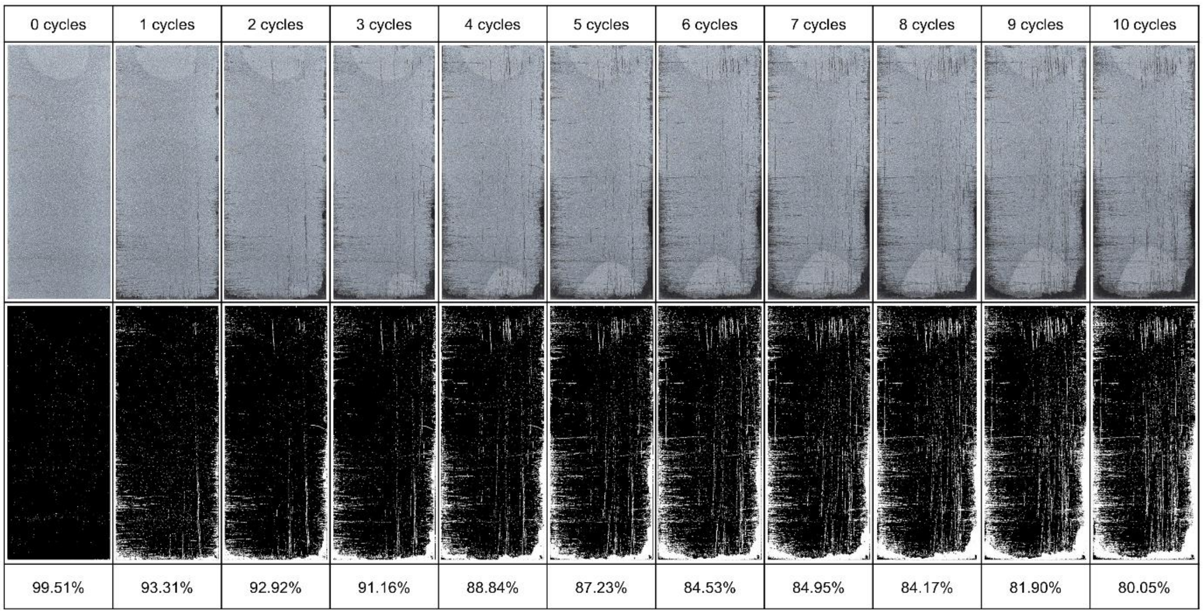

g) µ/n mix – Run No.1:

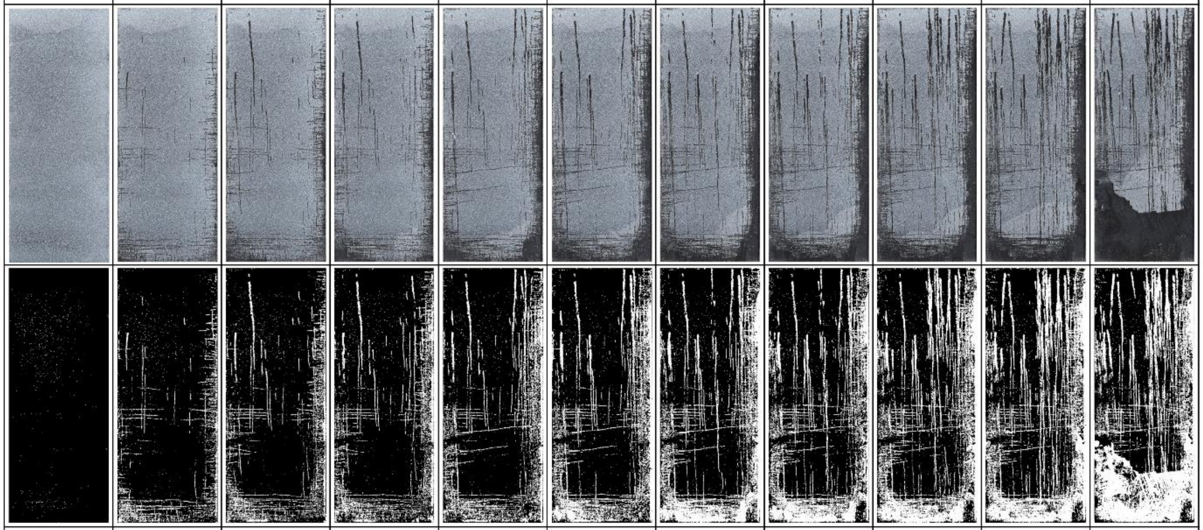

0 cycles	1 cycles	2 cycles	3 cycles	4 cycles	5 cycles	6 cycles	7 cycles	8 cycles	9 cycles	10 cycles
99.52%	91.58%	86.79%	84.24%	80.80%	76.69%	72.42%	71.85%	67.49%	62.45%	55.01%

h) µ/n mix – Run No.2:

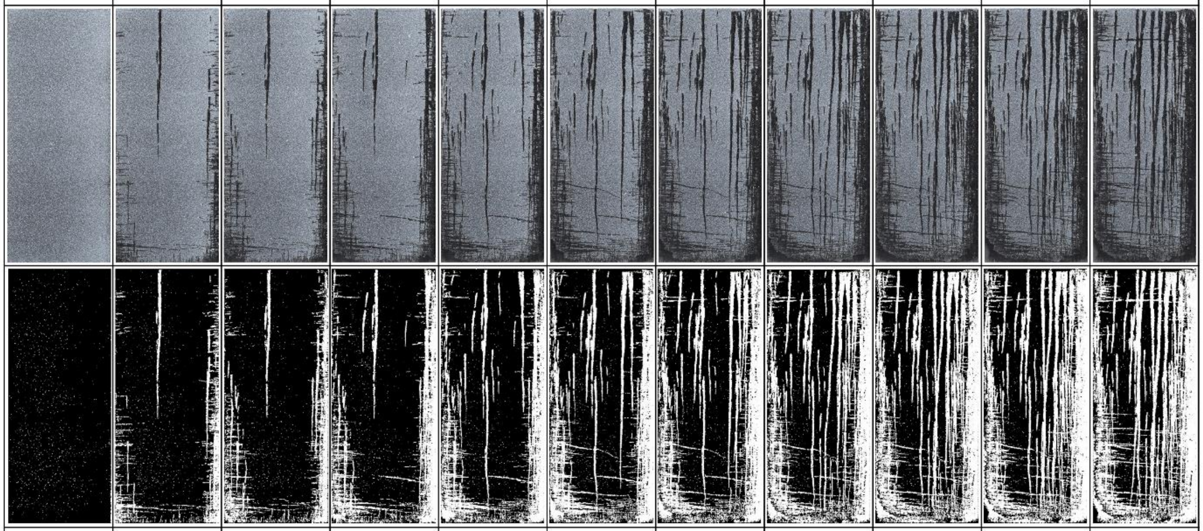

0 cycles	1 cycles	2 cycles	3 cycles	4 cycles	5 cycles	6 cycles	7 cycles	8 cycles	9 cycles	10 cycles
98.76%	88.66%	83.38%	77.85%	72.42%	66.73%	63.48%	59.31%	55.90%	53.70%	49.49%

i) µ/n mix – Run No.3:

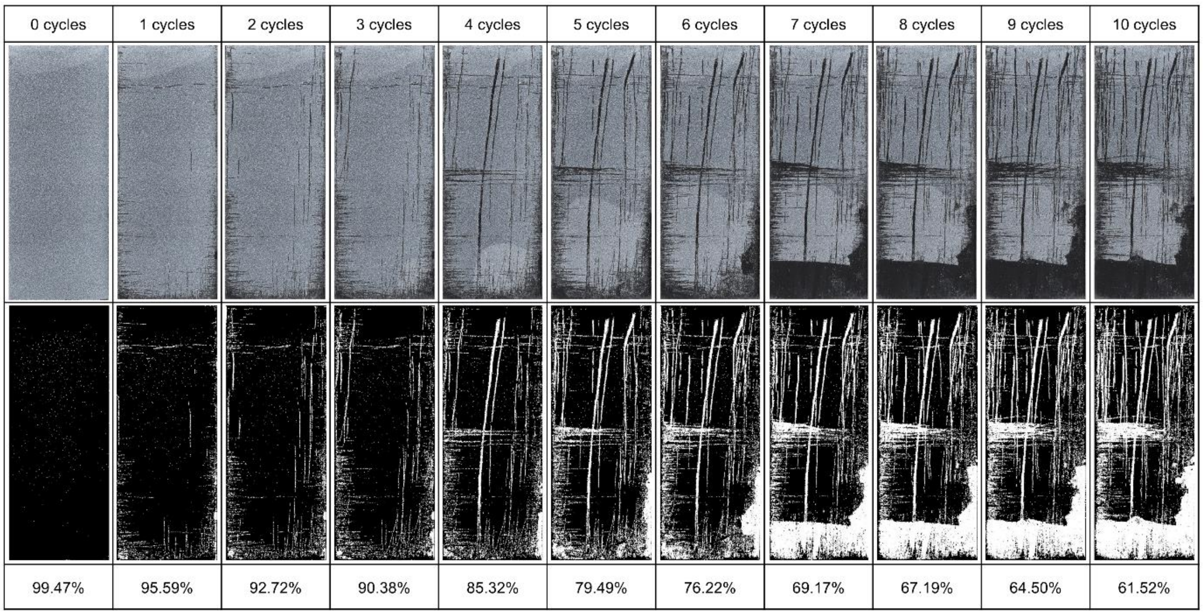

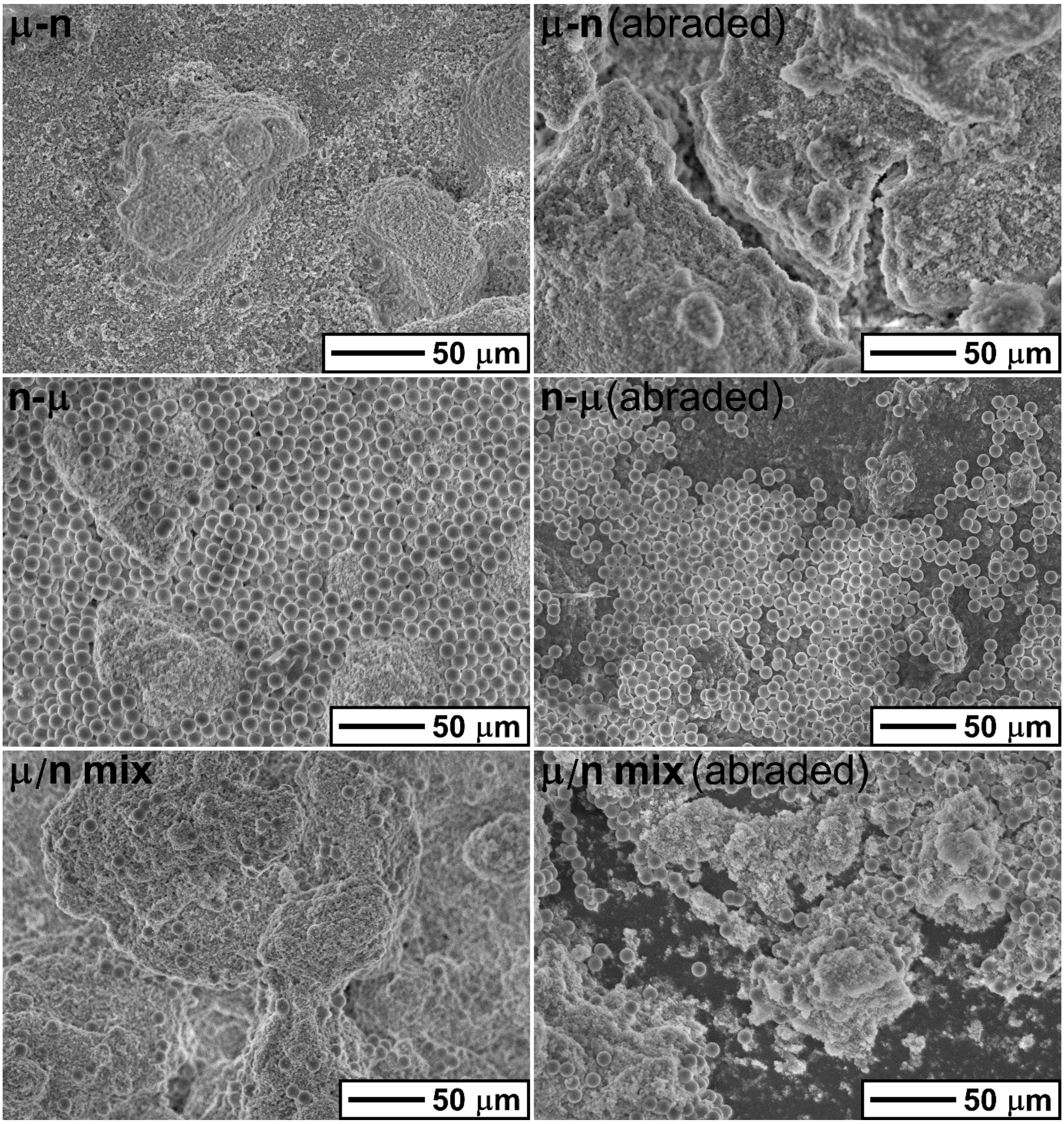

Figure SI13: SEM images of PDMS Sylgard 186 coatings made with μ-n, n-μ, and μ/n mix particle (as indicated). The coatings are as unabraded (left) and after ten abrasion cycles. Scale bars for each image are shown.
